# Whole genome transcriptomics reveals global effects including up-regulation of *Francisella* pathogenicity island gene expression during active stringent response in the highly virulent *Francisella tularensis* subsp. *tularensis* SCHU S4

**DOI:** 10.1099/mic.0.000550

**Published:** 2017-11-08

**Authors:** Amber L. Murch, Paul J. Skipp, Peter L. Roach, Petra C. F. Oyston

**Affiliations:** ^1^​ CBR Division, Defence Science and Technology Laboratory, Salisbury, UK; ^2^​ School of Chemistry, University of Southampton, Southampton, UK

**Keywords:** *Francisella tularensis*, pathogenicity islands, stringent response, transcriptional regulation, gene regulation

## Abstract

During conditions of nutrient limitation bacteria undergo a series of global gene expression changes to survive conditions of amino acid and fatty acid starvation. Rapid reallocation of cellular resources is brought about by gene expression changes coordinated by the signalling nucleotides' guanosine tetraphosphate or pentaphosphate, collectively termed (p)ppGpp and is known as the stringent response. The stringent response has been implicated in bacterial virulence, with elevated (p)ppGpp levels being associated with increased virulence gene expression. This has been observed in the highly pathogenic *Francisella tularensis* sub spp. *tularensis* SCHU S4, the causative agent of tularaemia. Here, we aimed to artificially induce the stringent response by culturing *F. tularensis* in the presence of the amino acid analogue l-serine hydroxamate. Serine hydroxamate competitively inhibits tRNA^ser^ aminoacylation, causing an accumulation of uncharged tRNA. The uncharged tRNA enters the A site on the translating bacterial ribosome and causes ribosome stalling, in turn stimulating the production of (p)ppGpp and activation of the stringent response. Using the essential virulence gene *iglC*, which is encoded on the *Francisella* pathogenicity island (FPI) as a marker of active stringent response, we optimized the culture conditions required for the investigation of virulence gene expression under conditions of nutrient limitation. We subsequently used whole genome RNA-seq to show how *F. tularensis* alters gene expression on a global scale during active stringent response. Key findings included up-regulation of genes involved in virulence, stress responses and metabolism, and down-regulation of genes involved in metabolite transport and cell division. *F. tularensis* is a highly virulent intracellular pathogen capable of causing debilitating or fatal disease at extremely low infectious doses. However, virulence mechanisms are still poorly understood. The stringent response is widely recognized as a diverse and complex bacterial stress response implicated in virulence. This work describes the global gene expression profile of *F. tularensis* SCHU S4 under active stringent response for the first time. Herein we provide evidence for an association of active stringent response with FPI virulence gene expression. Our results further the understanding of the molecular basis of virulence and regulation thereof in *F. tularensis*. These results also support research into genes involved in (p)ppGpp production and polyphosphate biosynthesis and their applicability as targets for novel antimicrobials.

## Introduction

*Francisella tularensis* is a highly virulent facultative intracellular bacterium, and the aetiological agent of tularaemia. This Gram-negative bacterium is able to infect a wide range of mammalian hosts, including humans with as few as 10 c.f.u. [1–3]. Three subspecies of *F. tularensis* are currently accepted: subspecies *tularensis* (type A) is found exclusively in North America [4], the less virulent subspecies *holarctica* (type B) is found in North America and Eurasia [4], and the relatively avirulent subspecies *mediasiatica* is found in central Asia [4]. A proposed fourth subspecies *novicida* currently remains a separate species despite its genetic similarities [5]. *F. tularensis* is a physically and genetically small micro-organism, comprising a single circular chromosomal genome of only 1.89 Mb and no virulence plasmids [2]. Despite this, the bacterium is able to invade and multiply within numerous cell types, with replication in mammalian macrophages at the core of *F. tularensis* pathogenesis. Following uptake, the bacterium resides temporarily within phagosomes, but then escapes to replicate in the cytoplasm, all the while evading the host immune system [3]. The molecular mechanisms controlling these processes are yet to be fully understood. However, many virulence genes have been identified following whole genome sequencing of *F. tularensis* strains and subspecies [6]. The *Francisella* pathogenicity island (FPI), which encodes a putative type VI secretion system, has been proven to be a major contributor to *Francisella* virulence [7, 8].

The 30 kb FPI encodes 17 genes, 11 of which comprise a type VI secretion system, and has been shown to be essential for survival and growth within host macrophages [7, 8]. The genomic region encoding the FPI is duplicated in the highly pathogenic *F. tularensis* subsp. *tularensis* and subsp. *holarctica* including the live vaccine strain [7]. Recent studies have shown that several genes encoded on the FPI are directly regulated by MglA, a protein that has been implicated in the stringent response of *Francisella* during starvation conditions and oxidative stress [9]. One such finding noted that the intracellular growth locus protein IglC was induced during intracellular infection of macrophages and was shown to be regulated by MglA [10]. However, little is currently understood about how *F. tularensis* regulates its molecular mechanisms for pathogenesis, with few regulatory proteins being revealed during whole genome analyses [11, 12]. The paucity of regulatory systems seems surprising due to the diversity of the cell types *F. tularensis* is able to infect, and the environments the bacterium can survive in (intracellular and extracellular) [5].

It has been frequently noted that bacterial stress response mechanisms are closely associated with virulence gene expression and bacterial survival in host cells [13, 14]. One such stress response pathway, the stringent response, has recently been implicated in *Francisella* virulence [11]. To survive and replicate in their environmental niche, bacteria must monitor and adapt to changing conditions by modifying their stress tolerance and nutrient utilization pathways [15]. During times of nutrient limitation bacteria are able to rapidly reallocate their cellular resources, by down-regulating processes such as nucleic acid and protein synthesis, and up-regulating essential amino acid production and protein degradation [16]. This synchronized sequence of events in response to amino acid and fatty acid starvation is known as the stringent response [16, 17]. The stringent response gives rise to a dramatic alteration of global gene expression profiles and is coordinated by the signalling nucleotides' guanosine tetraphosphate (ppGpp) and guanosine pentaphosphate (pppGpp), collectively termed (p)ppGpp [17], which accumulate under starvation conditions [18]. Global gene expression modifications are brought about by direct binding of (p)ppGpp to various targets, such as RNA polymerase and sigma factors [19]. Intracellular concentrations of (p)ppGpp in bacteria are modulated by two related enzymes: RelA and SpoT. RelA is a monofunctional (p)ppGpp synthetase and SpoT is a bifunctional (p)ppGpp synthetase/hydrolase [18]. RelA and SpoT have been shown to have activity in a range of bacteria including *Escherichia coli*, *Pseudomonas aeruginosa*, *Yersinia pestis* and *F. tularensis.* Both enzymes have been shown to synthesize (p)ppGpp from GDP or GTP and ATP in *E. coli* [20]. SpoT also degrades ppGpp into GDP and pyroposphate (PPi) and pppGpp to GTP and PPi, to prevent uncontrolled accumulation of (p)ppGpp [16, 17, 20]. Unabated accumulation of (p)ppGpp disrupts cell cycle control [17], therefore a degradation mechanism is essential for bacterial survival. Mutagenesis studies have found that it is possible to generate strains defective in RelA and RelA/SpoT, but inactivation of the *spoT* gene alone is often a lethal mutation [21].

Work conducted previously has investigated the role of RelA in *Francisella* virulence and intracellular survival. It was found that inactivation of the *relA* gene in *Francisella novicida* resulted in a mutant that was unable to produce (p)ppGpp under amino acid starvation conditions [11]. When tested in a murine model of tularaemia, the mutant was attenuated, and induced protective immunity to the virulent wild-type organism, demonstrating the importance of (p)ppGpp for pathogenesis of *F. novicida* [22].

**Fig. 1.**
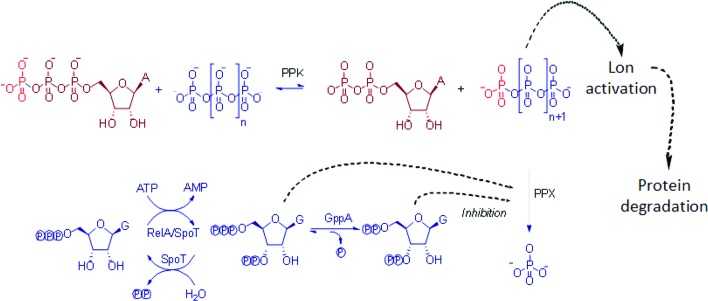
Interaction of (p)ppGpp biosynthesis with polyphosphate metabolism. PPK catalyses the reversible synthesis of polyphosphate from the terminal phosphate from ATP [46]. PPX catalyses the degradation of polyphosphate into free Pi residues [47, 48]. GppA, an exopolyphosphatase, catalyses the conversion of pppGpp to ppGpp [47, 49]. RelA catalyses the synthesis of (p)ppGpp from ATP and GTP or GDP [50–52]. SpoT catalyses both the synthesis of (p)ppGpp from ATP and GTP or GDP and the degradation of ppGpp to GDP and PPi and pppGpp to GTP and PPi [53]. Polyphosphate also activates the Lon protease which leads to the degradation of ribosomal proteins to increase the pool of available amino acids for protein synthesis when more favourable conditions return [23].

Regulatory systems such as the stringent response rarely act alone and often form a hierarchy of control with overlapping regulons to create a network of biochemical pathways to allow fine tuning of responses. An example of this interaction of regulatory pathways has been reported between the stringent response and inorganic polyphosphate metabolism (Fig. 1) [23].

Inorganic polyphosphate has been shown to have a role in both the stringent response and bacterial virulence, and levels of polyphosphate in bacterial cells are regulated by a polyphosphate kinase, encoded by *ppk* and an exopolyphosphatase, encoded by *ppx* [24, 25]. Polyphosphate accumulates alongside (p)ppGpp during stringent conditions due to the inhibition of PPX by (p)ppGpp [23, 26–28]. Polyphosphate is also involved in activating the Lon protease complex which degrades ribosomal proteins to provide a pool of amino acids for the synthesis of new proteins, a key step in preparation for stationary phase survival, and a key component of the stringent response [29].

Previous work has found that mutation of *Francisella ppk* caused defects for intracellular growth in macrophages and attenuation in mice, supporting a key role for the putative polyphosphate kinase in *Francisella* virulence [30]. Inactivation of the gene annotated as FTN1472/FTT1564 resulted in the abolishment of polyphosphate production in *F. novicida*. Stringent response mutants which have diminished levels of (p)ppGpp have also been found to harbour lower levels of polyphosphate [22, 28], suggesting polyphosphate plays a key role in stress survival.

In order to simulate stringent response conditions *in vitro*, artificial induction of the stringent response is possible using the amino acid analogue l-serine hydroxamate. Serine hydroxamate is a competitive inhibitor of seryl transfer ribonucleic acid (tRNA) synthetase [31]. l-serine hydroxamate will compete with l-serine, excluding the amino acid substrate from the enzyme, leading to depletion of seryl-tRNA. This prevents charging of tRNA [31, 32] and if accumulated to sufficient levels, the uncharged tRNA will enter the bacterial ribosome at the A site. This causes ribosome stalling, in turn activating ribosome-associated RelA, and subsequent synthesis of (p)ppGpp [33].

To investigate the regulatory networks involved in the stringent response and polyphosphate metabolism, global gene expression profiles can be elucidated. Until recently, microarray hybridization was the preferred technique for evaluating whole genome expression patterns. However, limitations associated with this technique, such as the requirement for *a priori* knowledge of the genome sequence under interrogation and the time and costs involved, have led to its gradual replacement by high-throughput sequencing technologies. High-throughput sequencing offers greater resolution, no prerequisite for known genome sequences and the ability to analyse multiple samples in a massively parallel fashion. Thus, RNAseq was selected for analysis of the *Francisella* stringent regulon in this study.

## Results and discussion

### Serine hydroxamate titration

**Fig. 2.**
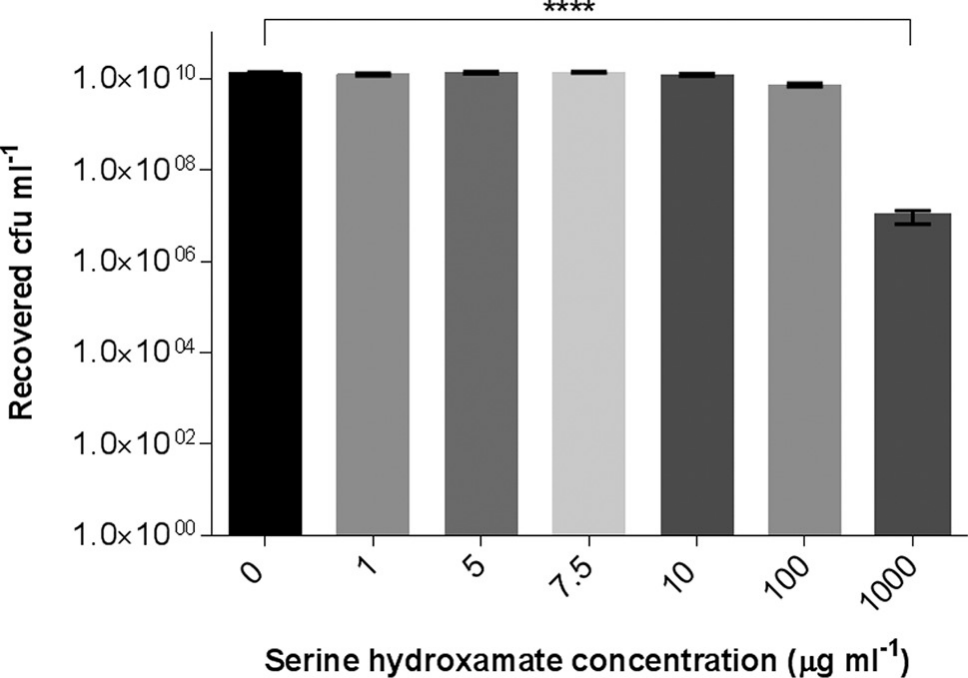
Serine hydroxamate titration to establish the concentration at which growth of *F. tularensis* SCHU S4 is inhibited. Statistical significance was determined by one-way ANOVA at a 95 % confidence interval with Bonferroni’s correction for multiple comparisons. (**** – *P*<0.0001).

The stringent response was induced using a low concentration of serine hydroxamate, sufficient to induce the stringent response, whilst not inhibiting the growth of *F. tularensis* SCHU S4 in Chamberlain’s defined medium (CDM) without dl-serine. A titration was initially carried out to establish the lowest concentration of serine hydroxamate that could be added to cultures without inhibiting growth, but which has been shown in previous studies to trigger the stringent response (Fig. 2). Due to this study focussing on the highly pathogenic intracellular bacterium, *F. tularensis*, subspecies *tularensis* and the experimental work being carried out at biosafety level three, it was not possible to quantify (p)ppGpp levels in serine hydroxamate-treated *F. tularensis* cultures. However, previous studies and in-house experimental data had shown that the conditions used in this work were sufficient to induce the stringent response [11, 34].

The addition of up to 100 µg ml^−1^ serine hydroxamate had no significant effect on the growth of *F. tularensis* SCHU S4. Addition of 1000 µg ml^−1^ serine hydroxamate had a toxic effect, significantly reducing the number of viable bacteria recovered from cultures, possibly inducing more general stress responses as well as the stringent response. Nearly three orders of magnitude fewer viable bacteria were recovered from cultures grown in the presence of 1000 µg ml^−1^ serine hydroxamate. Consequently, it was decided that cultures containing concentrations of between 0 and 100 µg ml^−1^ serine hydroxamate would be analysed by reverse-transcriptase PCR (RT-PCR) for virulence gene expression.

### *iglC* expression under amino acid starvation conditions

**Fig. 3.**
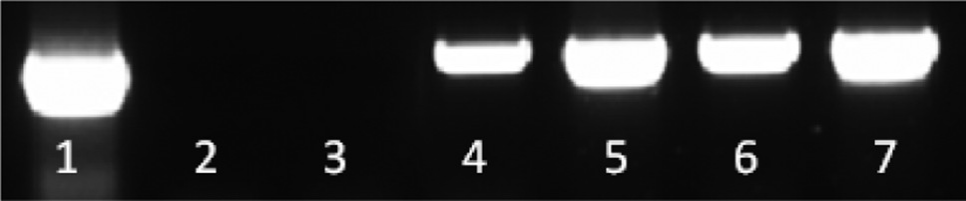
Expression of *iglC* in *F. tularensis* SCHU S4 can be induced by supplementing growth media with serine hydroxamate as determined by RT-PCR. Lane 1, mouse β-actin gene control amplified from mouse liver total RNA; lane 2, no DNA control; lane 3, cDNA from 0 µg ml^−1^ serine hydroxamate; lane 4, cDNA from 1 µg ml^−1^; lane 5, cDNA from 10 µg ml^−1^; lane 6, cDNA from 50 µg ml^−1^; lane 7, cDNA from 100 µg ml^−1^.

To establish the concentration of serine hydroxamate required to initiate the stringent response, RT-PCR targeting the *iglC* gene on the FPI was used to determine if the FPI genes were being expressed, as a marker of stringent response activation [35, 36]. The FPI-encoded virulence gene *iglC* was selected as a molecular indicator of active stringent response due to work by Charity *et al.* who showed that all FPI genes are up-regulated during the stringent response under the control of (p)ppGpp and the global regulators MglA and SspA [36]. Primers iglCrtpcrF and iglCrtpcrR (Table S2, available in the online Supplementary Material) were used to assess *iglC* expression (557 bp) and mouse β-actin gene (540 bp) was used as a control gene with mouse total liver cDNA. A dilution series of serine hydroxamate concentrations were tested to establish the lowest concentration at which *iglC* expression could be observed. RT-PCR showed that *iglC* was induced in the presence of very low concentrations of serine hydroxamate (1 µg ml^−1^), and was not expressed in media without serine hydroxamate supplementation (Fig. 3).

Following RT-PCR, RNA sequencing was used to analyse the global gene expression profiles of *F. tularensis* cultures supplemented with 1 and 10 µg ml^−1^ serine hydroxamate, compared to a control culture containing no serine hydroxamate. 1 µg ml^−1^ serine hydroxamate was selected as the lowest concentration at which the stringent response was expected to be switched on, and 10 µg ml^−1^ serine hydroxamate was selected as a concentration 10-fold higher, to determine if more significant and widespread global gene expression effects could be observed. Neither concentration of serine hydroxamate selected for RNAseq inhibited the growth of *F. tularensis*.

### *F. tularensis* global gene expression profile

**Fig. 4.**
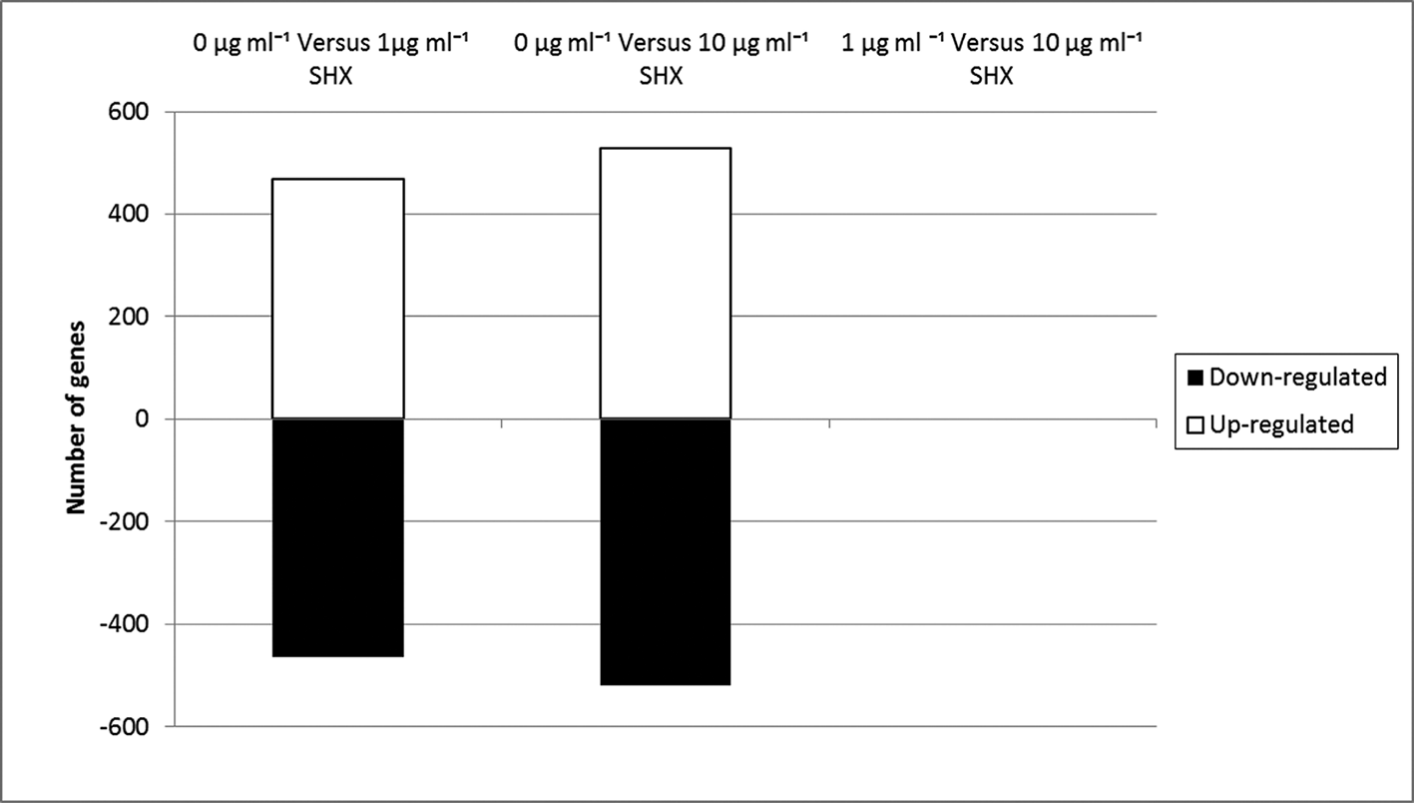
Total number of significantly differentially expressed genes in each serine hydroxamate condition tested. A total of 1005 (60.80 % of *F. tularensis* total genes) genes showed changes in expression in the comparison between 0 and 1 µg ml^−1^ serine hydroxamate treatments, and a total of 1089 (65.88 % of total genes) genes showed changes in expression in the comparison between 0 and 10 µg ml^−1^ serine hydroxamate.

**Fig. 5.**
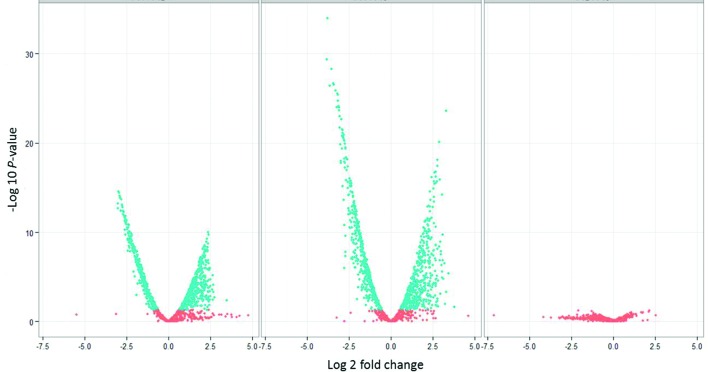
Volcano plots showing significantly differentially expressed genes derived from analysis at a 95 % confidence interval. Data points in blue are significant, points in red are not significant. The far left image represents gene expression in the 0 versus 1 µg ml^−1^ serine hydroxamate condition. The middle image represents gene expression in the 0 versus 10 µg ml^−1^ serine hydroxamate condition and the far right image represents gene expression in the 1 µg ml^−1^ versus 10 µg ml^−1^ serine hydroxamate condition.

The gene expression profiles obtained from *F. tularensis* cultures treated with 1 µg ml^−1^ serine hydroxamate revealed a total of 1005 (60.80 % of *F. tularensis* total genes) genes showing changes in expression compared to the control culture with no serine hydroxamate (Fig. 4). The expression profiles from cultures treated with 10 µg ml^−1^ serine hydroxamate revealed a total of 1089 (65.88 % of total genes) genes showing changes in expression compared to the control culture (Fig. 4). Of those, 219 genes showed more than twofold expression changes in the comparison between conditions 0 and 1 µg ml^−1^, and 316 genes showed more than twofold expression changes between 0 and 10 µg ml^−1^. The most significantly differentially expressed genes between 0 and 1 µg ml^−1^ serine hydroxamate-treated conditions are listed in Table S1. Interestingly, there were no genes that passed the significance filters for differential expressed between 1 and 10 µg ml^−1^ serine hydroxamate as shown by the graph in Fig. 4 and the volcano plots in Fig. 5. This indicated that increasing the concentration of serine hydroxamate did not significantly increase the global effects of the stringent response on gene expression, but was also consistent with the observations made from the RT-PCR experiments (Fig. 3). However, the volcano plots in Fig. 5 did reveal statistically significant differences between the high and low serine hydroxamate conditions compared to the untreated control condition. This indicated that increasing the concentration of serine hydroxamate did not significantly increase the global effects of the stringent response on gene expression, but was also consistent with the observations made from the RT-PCR experiments (Fig. 3). Although gene expression differences were uncovered in this work, a potential drawback of using transcriptomics to evaluate gene expression changes on a global scale is that if transcript levels of certain proteins are particularly low or show narrow differences in expression levels between conditions, these could potentially be filtered out of the overall analysis due to a lack of significant data. However, this technique does offer many advantages in terms of accuracy of defining transcript levels and coverage of the genome. Although this advantage is dependent on the comprehensiveness of reference sequence availability, and the annotation of the *F. tularensis* reference genome sequence remains incomplete in many regions. This being said, these results suggested that, in the range of serine hydroxamate concentrations studied, the stringent control of gene expression in response to stress was very much an on/off response at the point of initiation as opposed to a gradual adaptive process. However it was demonstrated that higher concentrations of serine hydroxamate led to more significant and widespread changes. This could indicate that after the stringent response has been triggered the response can further adapt in response to environmental cues.

**Fig. 6.**
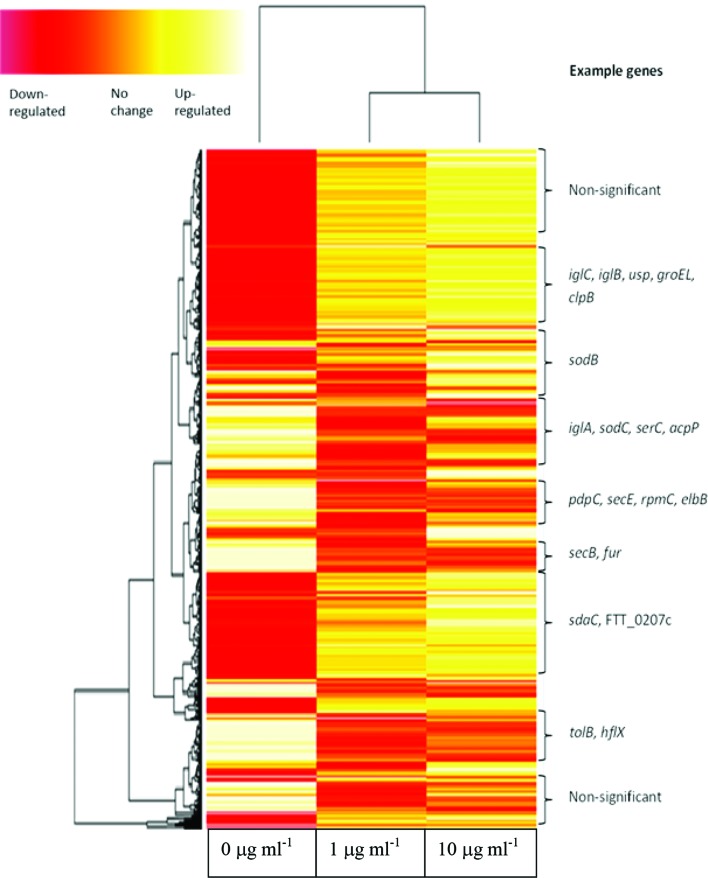
Heat map showing global gene expression profiles of *F. tularensis* SCHU S4 isolated from cultures treated with different concentrations of serine hydroxamate. The far left profile corresponds to the control 0 μg ml^−1^ serine hydroxamate condition, the middle profile corresponds to the 1 µg ml^−1^ serine hydroxamate condition and the far right profile corresponds to the 10 µg ml^−1^ serine hydroxamate condition. Yellow bands indicate increased gene expression levels, and red bands indicate decreased expression levels. Individual genes are indicated on the *y-*axis and clustered according to expression level in the different serine hydroxamate conditions. This shows only examples of genes in the different clusters as it is not possible to annotate every single gene in this figure. Each horizontal line denotes a separate *F. tularensis* gene.

Hierarchical cluster analysis was used to generate a heat map of the global gene expression profile of *F. tularensis* cultured in the presence of either 0, 1 or 10 µg ml^−1^ serine hydroxamate which was used to induce the stringent response and associated gene expression changes (Fig. 6). Hierarchical clustering of differential gene expression data designated the ‘no serine hydroxamate’ control group as the outlier and the two serine hydroxamate-treated conditions as a clustered group in the distance matrix tree (Fig. 6). Genes were clustered according to expression level, and it was apparent that the significance of differential gene expression generally increased the higher the concentration of serine hydroxamate used. By increasing the concentration of serine hydroxamate, global gene expression changes become slightly more widespread and significant when compared to the control culture without serine hydroxamate supplementation. However, there was little difference in gene expression levels between the two serine hydroxamate-treated conditions as demonstrated by the lack of statistically significant differences between these conditions (Fig. 5).

**Fig. 7.**
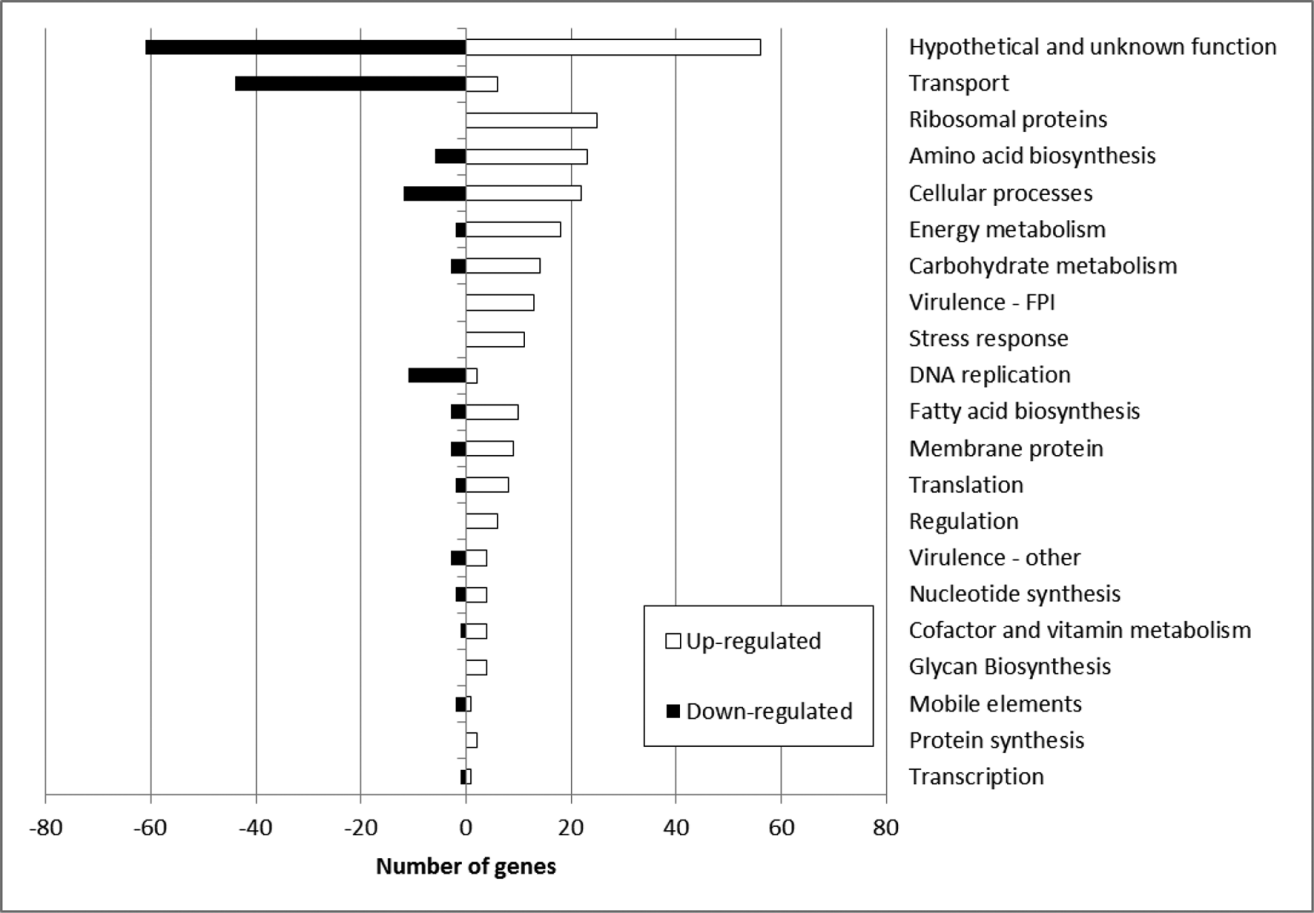
Number of genes up-regulated or down-regulated from the top 400 most significantly differentially expressed genes in the 1 µg ml^−1^ serine hydroxamate condition, classified by functional category.

It was also apparent that expression levels of genes which belonged to particular functional groups were collectively up-regulated or down-regulated in the serine hydroxamate-treated samples. Fig. 7 shows the distribution of genes allocated to various functional categories and their associated expression level. Although categories such as virulence genes and transport showed clear up- or down-regulation patterns (fold change shown in the Supplemental Material), many genes were categorized as hypothetical proteins or with unknown function, which highlighted the need for more studies into characterizing the *F. tularensis* genome.

### Virulence gene expression

**Fig. 8.**
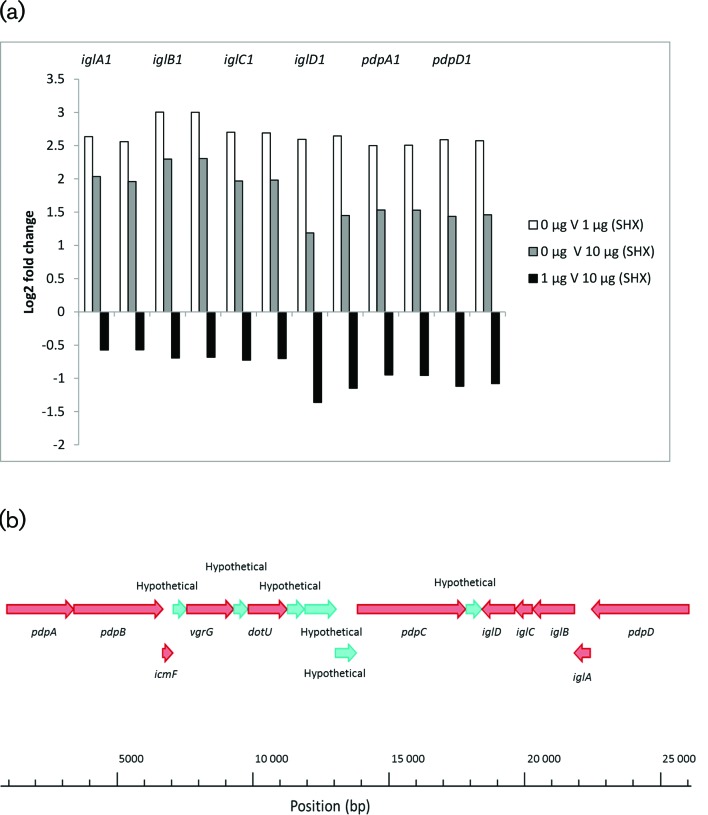
(a, b) Log_2_-fold change in gene expression levels of genes encoded on the FPI in the different serine hydroxamate conditions tested (a). Gene arrangement of the FPI which encodes a putative TSSS (b).

Whole genome transcriptomics of serine hydroxamate-treated *F. tularensis* samples revealed a significant up-regulation of virulence-associated genes, particularly those encoded on the FPI. Previous research has suggested an association of virulence gene expression and the stringent response in *Francisella* [36]. The genes *iglA1, iglA2, iglB1, iglB2, iglC1, iglC2, iglD1, iglD2, pdpA1, pdpA2, pdpD1* and *pdpD2* encoded on the FPI showed a significant increase of at least 2.5-fold change and a significance score of at least 2.75E-09 in expression level in the active stringent response cultures of *F. tularensis* compared to the untreated control culture (Fig. 8). This supports previous observations by Charity *et al*., who noted a significant decrease in expression levels of FPI virulence genes, including the intracellular growth locus genes and the pathogenicity determinant proteins in Δ*mglA* and Δ*sspA* mutant backgrounds compared to wild-type *Francisella* [35]. That study showed that, without the regulatory protein, MglA (in cooperation with SspA), expression levels of FPI genes cannot be sufficiently regulated [35]. Wehrly *et al*. reported an up-regulation of *Francisella* virulence determinants inside macrophages, which would be expected to be an environment that would activate the stringent response due to a lack of nutrient availability and an abundance of reactive oxygen species [37]. Wehrly *et al*. also reported FPI gene expression rapidly increased within the first hour post-infection, and then reached maximum expression levels by the end of the cytosolic replication stage of infection, approximately 12–16 h post infection [37]. They also found that *iglC* expression was much higher than other FPI genes whereas *pdpC* was significantly down-regulated [37]. As previous research has shown gene expression during stationary phase to be most representative of gene expression during the stressful conditions associated with *in vivo* survival, it was anticipated that serine hydroxamate treatment would simulate starvation conditions and result in similar gene expression profiles. As expected, gene expression analysis presented herein also found *iglC1* and *iglC2* to show high expression levels compared to some other FPI-encoded genes such as *pdpC1* and *pdpC2*, however it was also found that *iglB1* and *iglB2* showed comparable expression levels, conversely to Wehrly *et al*. Additionally, in support of the observation that Wehrly *et al*. made that expression of FPI genes increased rapidly in the initial stages of infection then decreased after 16 h, analysis from this study showed that expression levels of the FPI genes were higher in the 1 µg ml^−1^ serine hydroxamate condition compared to the 10 µg ml^−1^ serine hydroxamate condition. This result could indicate that increasing the concentration of serine hydroxamate mimics a later stage of the infection lifecycle of *F. tularensis*. However, as it is known that high concentrations of serine hydroxamate inhibit bacterial growth, perhaps this result reflects mRNA degradation following rapid increase in mRNA transcript levels during the initial infection. In addition to the evident contribution of FPI genes to *Francisella* virulence, various metabolic pathways have also been shown to contribute to the pathogenesis of this micro-organism. One such pathway, which remains relatively unstudied, is the glycine cleavage system (GCS) [38]. This system facilitates the degradation of glycine to acquire 5,10-methylene-tetrahydrofolate, a one carbon donor utilized in the production of serine, thymidine and purines. This pathway contributes to pathogen fitness *in vivo*, where metabolites such as serine are limited [38]. As such, it has been reported that homologues of the GCS are up-regulated during *F. tularensis* infection of macrophages [37]. A *gcvH* homologue was discovered to be strongly induced in *Francisella* isolated from mouse spleens [39]. In support of this finding, *gcvH* was the most significantly up-regulated gene in our comparison between 1 and 10 µg ml^−1^ serine hydroxamate conditions, reported herein (Table S1). Further evidence for the importance of the GCS has been reported by Brown *et al*., where *gcvT* was required for full *in vivo* virulence of *F. tularensis* following investigation of this pathway using deletion mutants lacking *gcvT* [38]. Studies of this deletion mutant also revealed a requirement of the GCS in *F. tularensis* SCHU S4 in serine limiting conditions in broth, however had no effect on the survival of *F. tularensis* in rich media in macrophages or lung epithelial cells. However intracellular growth assays performed in minimal media, depleted for serine, intracellular growth defects were apparent in *F. tularensis* strains lacking a functional *gcvT* homologue. These findings by Brown *et al*. indicated that culture conditions reported in the work presented herein potentially represented serine starvation conditions that *F. tularensis* might encounter in the host environment.

### Stress response gene expression

In addition to virulence gene expression, significant differences were observed in expression of stress response genes, particularly those involved in responding to oxidative stress. The genes encoding the superoxide dismutases *sodB* and *sodC* were among the most significantly up-regulated genes in this study (Table S1). Additionally, the universal stress protein *usp* showed a 2.7-fold change increase in expression level in serine hydroxamate-treated cultures. Other stress response genes such as *groEL* and *clpP* which had previously been identified as being up-regulated in studies of *Francisella* gene expression in macrophages [37] also showed increased expression in the starved culture conditions in our study.

### Metabolic gene expression

As expected during stringent conditions, many processes such as cell division and DNA replication are significantly down-regulated in order to conserve cellular resources for survival during stationary phase. However, bacterial cells undergoing the stringent response will also up-regulate processes in preparation for when nutrient availability improves. Such processes include metabolic pathways for the synthesis of amino acids, fatty acids and energy production. FTT1666c, a 3-hydroxyisobutyrate dehydrogenase, a protein involved in amino acid metabolism, showed the third most significant increase in expression in the 1 µg ml^−1^ serine hydroxamate condition. This gene was previously shown by Charity *et al*. to be significantly down-regulated in Δ*mglA* and Δ*sspA* mutant backgrounds compared to wild-type *Francisella*, implying that FTT1666c could be an important virulence determinant in *F. tularensis* as MglA, in cooperation with SspA is a known regulator of virulence gene expression in *F. tularensis*. SdaA, another protein involved in amino acid production also showed a 2.5-fold increase in expression in the 1 µg ml^−1^ serine hydroxamate condition. PepA, SerC and FTT1253 also showed at least a 2.5-fold increase in their expression levels in the 1 µg ml^−1^ serine hydroxamate condition.

### Regulatory gene expression

Bacterial gene expression is mediated by proteins, and more recently established small regulatory RNAs that either act on a global scale or at specific sites in the genome to either activate or repress gene expression. Regulatory systems in *F. tularensis* remain poorly characterized, with the majority of two-component regulatory system components being identified as orphans [40]. Well-characterized regulatory proteins such as sigma factors are generally not affected by environmental conditions and maintain a constant basal level of expression regardless of stressful environmental conditions. For example, as reported in Table S1, *rpoD* (σ70) did not show a significant difference in its expression level in any of the conditions in this experiment. Conversely, genes from two-component regulatory systems, which are involved in sensing environmental conditions, such as FTT1557c a two-component response regulator were among the most significantly up-regulated genes in the serine hydroxamate-treated samples. Previous research has demonstrated that targeted deletion of the gene *fevR,* which encodes a transcriptional regulator, causes attenuation of *F. tularensis* SCHU S4 in a murine model of infection and is unable to survive or proliferate in macrophages [37]. In addition, this gene showed a significant decrease in expression in a Δ*mglA* and Δ*sspA* mutant background compared to wild-type *Francisella*, which implied that FevR could be an important virulence determinant in *F. tularensis.* The conserved RNA chaperone, Hfq, has recently been implicated in stringent response regulation, whereby RelA facilitates binding of low affinity RNAs to Hfq to enable gene expression changes in response to starvation conditions [41]. *Hfq* was found to be among the most significantly up-regulated genes in both serine hydroxamate-treated cultures. However, the stringent response gene *relA* did not reveal a significant difference in its expression level, whereas *spoT* showed a significant increase in expression level in the starved *Francisella* cultures (Table S1).

The serine hydroxamate-induced stringent response leads to the accumulation of (p)ppGpp and the eventual downstream metabolism of (p)ppGpp requires the pyrophosphate hydrolase activity of SpoT. Our working model for the expression levels of RelA and SpoT during the stringent response included a basal level of unactivated RelA present in bacteria which could be activated in response to amino acid deficiency-induced ribosome stalling. The synthesized (p)ppGpp resulted in global changes in expression levels, including up-regulation of SpoT (Table S1). The (p)ppGpp synthetase activity of SpoT can supplement the activity of RelA in response to nutrient deficiency, but if nutrient levels are restored, the SpoT (p)ppGpp pyrophosphate hydrolase activity can participate in restoring (p)ppGpp levels to the resting state (lower) level, thus switching off the stringent response.

### RT-PCR validation of RNA-seq observations

**Fig. 9.**
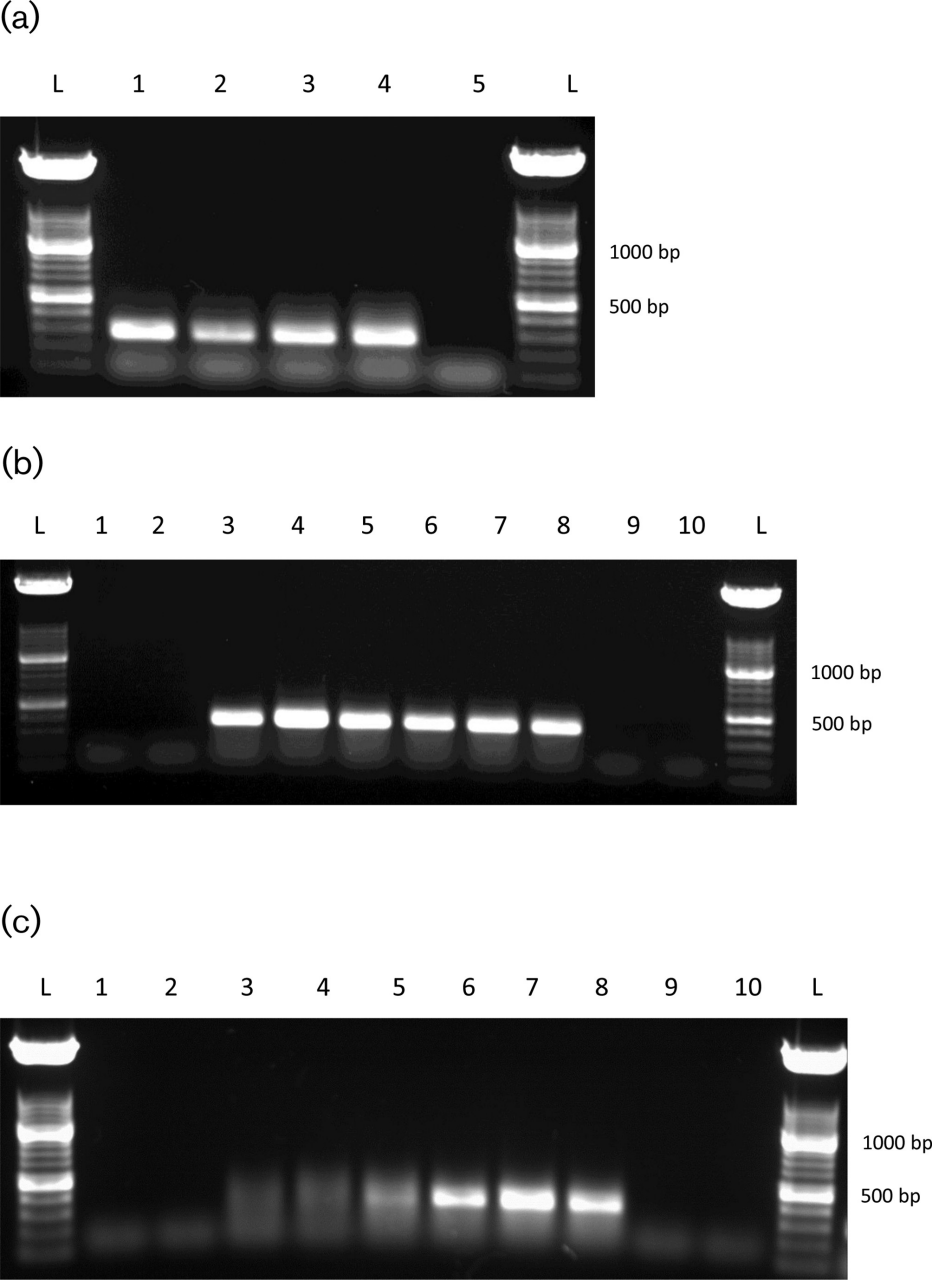
(a–c) Expression of the 16S rRNA housekeeping gene (a), FTT0613 (350 bp product) (b) and FTT1334 (350 bp product) (c) in *F. tularensis* SCHU S4 grown in media supplemented with or without serine hydroxamate as determined by RT-PCR. Lane 1, cDNA from 0 µg ml^−1^ serine hydroxamate; lane 2, cDNA from 1 µg ml^−1^ serine hydroxamate; lane 3, cDNA from 10 µg ml^−1^ serine hydroxamate; lane 4, cDNA from 100 µg ml^−1^ serine hydroxamate; lane 5, no cDNA control.

Two genes that showed significant up-regulation in serine hydroxamate-treated cultures of *F. tularensis* were selected for RT-PCR validation of the aforementioned differential expression. FTT0613 and FTT1334 were the top ranking differentially expressed hypothetical proteins selected from the dataset comparing *F. tularensis* treated with 0 µg ml^−1^ serine hydroxamate and *F. tularensis* treated with 1 µg ml^−1^ serine hydroxamate. Primers 0613rtpcrF/0613rtpcrR and 1334rtpcrF/1334rtpcrR (Table S2) were used to amplify 350 and 382 bp products, respectively, to determine expression levels of FTT0613 and FTT1334 in the serine hydroxamate-treated *F. tularensis* cultures. 16S rRNA was selected as a stable reference gene to which expression levels of the target genes could be compared. Primers 16SrtpcrF/16SrtpcrR (Table S2) were used to amplify a 287 bp product targeting the 16SrRNA gene. The 16S rRNA reference gene showed stable expression levels across all serine hydroxamate conditions tested (Fig. 9a), whereas FTT0613 showed no expression in *F. tularensis* cultured without serine hydroxamate and stable expression in those cultures treated with 1, 10 and 100 µg ml^−1^ serine hydroxamate (Fig. 9b). FTT1334 also revealed no expression in the 0 µg ml^−1^ serine hydroxamate condition, low expression levels at 1 µg ml^−1^ serine hydroxamate and higher expression levels at 10 and 100 µg ml^−1^ serine hydroxamate (Fig. 9c).

### Proteomic profiling of *F. tularensis* under stringent response activation

**Fig. 10.**
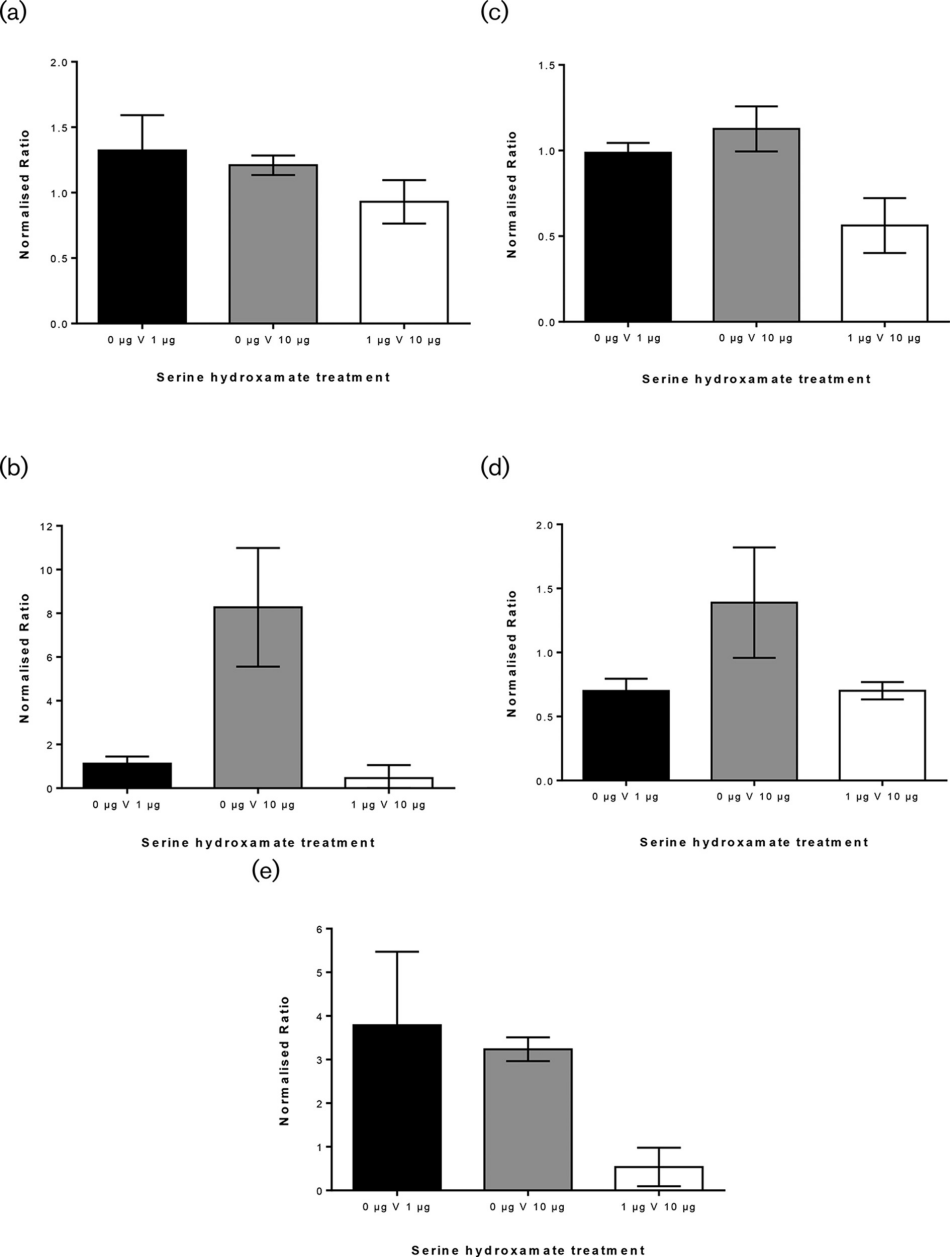
(a–e) Protein levels of IglC, UbiE, Hfq, Usp and SspA determined from whole proteome mass spectrometry, comparing ratios derived from the total *F. tularensis* SCHU S4 protein isolated from the cultures treated with 1 µg ml^−1^ of serine hydroxamate. Cultures treated with serine hydroxamate IglC showed a 1.27-fold increase in expression, however this was not statistically significant as determined by one-way ANOVA (*P*=0.0989). UbiE showed an 8.35-fold increase in expression in cultures treated with serine hydroxamate, which was statistically significant as determined by one-way ANOVA (*P*=0.0018). Hfq showed a 1.2-fold increase in expression in cultures treated with serine hydroxamate, which was statistically significant as determined by one-way ANOVA (*P*=0.8485). Usp showed a 1.4-fold increase in expression in cultures treated with serine hydroxamate, which was statistically significant as determined by one-way ANOVA (*P*=0.0259). SspA showed a 3.8-fold increase in expression in cultures treated with serine hydroxamate, which was statistically significant as determined by one-way ANOVA (*P*=0.0166).

Mass spectrometry was performed on prepared protein lysates of *F. tularensis* cultures grown in active stringent response conditions using the same serine hydroxamate concentrations as the previously discussed gene expression analysis (0, 1 or 10 µg ml^−1^ serine hydroxamate). Lysates were either inactivated at 60 or 100 °C for subsequent proteomic analysis. Proteomic profiling was selected as an appropriate technique to complement gene expression profiling results. However it should be noted that there are fundamental biological and practical differences between the two techniques which give rise to apparent discrepancies between datasets, due to various factors reviewed by Gandhi *et al.* [42] including but not limited to: post-transcriptional and post-translational regulation, variations in protein abundance and stability, and the fact that mRNA levels do not necessarily correlate with protein activity or abundance [42, 43]. However, taking these challenges into account, inferences can be made about the proteomic profile of *F. tularensis* in different stress-inducing conditions. Of the predicted protein coding sequences, 1804 ORFs, 1104 proteins (61 %) were detected in this screen. When the *F. tularensis* proteome of the 0 µg ml^−1^ serine hydroxamate condition was compared to the 10 µg ml^−1^ serine hydroxamate condition, 14 proteins showed a greater than twofold decrease and 25 proteins showed a greater than twofold increase in the levels. This finding is somewhat different to the transcriptomics data which revealed 1005 (60.80 % of the total *F. tularensis* genes) and a total of 1089 (65.88 % of the total genes) genes showed significant changes in expression in the comparison between 0 and 1 µg ml^−1^ serine hydroxamate and 0 and 10 µg ml^−1^ serine hydroxamate treatments. Proteins that showed an increase in expression in the comparison between 0 and 10 µg ml^−1^ serine hydroxamate treatments included IglC1 (FTT_1357 c), Q5NEC5, which is an intracellular growth locus protein which resides on the FPI, showing a 1.27-fold increase in expression, however this was not statistically significant (Fig. 10a). UbiE (FTT_1296), Q5NFE1, is a methyltransferase involved in ubiquinone biosynthesis and showed an 8.35-fold increase in expression, one of the highest and statistically significant expression levels observed in the dataset (Fig. 10b). Hfq (FTT_0630), Q5NH41, which binds RNAs facilitated by the stringent response protein RelA showed a 1.2-fold increase in expression, although this was not statistically significant (Fig. 10c). Stress response proteins that also showed a correlating increase in expression in the proteomic and transcriptomic datasets were Usp (FTT_0245), Q5N144, the universal stress protein and SspA (FTT_0458), Q5NHJ6, the stringent starvation protein (Fig. 10d, e respectively). Whereas SodB (FTT_0068), Q5NIJ9, a super oxide dismutase, showed no significant change in expression when comparing 0 and 10 µg ml^−1^ serine hydroxamate treatments, a 1.32-fold increase was however shown in expression when comparing 0 and 1 µg ml^−1^ serine hydroxamate treatments. Contrary, however, to the transcriptomics and RT-PCR results, the hypothetical proteins FTT1334 and FTT0613 did not show a significant change in expression levels in the proteomic results when comparing serine hydroxamate-treated samples to the control. This discrepancy highlights the challenges associated with integrating transcriptomic and proteomic data.

This study has demonstrated the direct association of *Francisella* virulence gene expression with an active stringent response, triggered by amino acid starvation conditions. In this study we demonstrated the ability to induce the stringent response artificially by culturing *F. tularensis* in the presence of low concentrations of the amino acid analogue serine hydroxamate. This allowed the stringent response to be studied *in vitro* at the global gene expression level by high-throughput technologies such as RNA-seq. Using the *iglC* virulence gene as a genetic marker of active stringent response, it was anticipated that upon artificial amino acid starvation by the addition of serine hydroxamate, *iglC* expression would be switched on or up-regulated. This was first demonstrated by RT-PCR, and then confirmed by whole genome transcriptomics. Whole genome transcriptomics also revealed differential expression of the other genes comprising the type VI secretion system on the FPI and other genes involved in *Francisella* virulence such as *groEL* and *dsbA*. It has previously been established in *Y. pestis* that bacterial cells in stationary phase of growth, *in vitro*, show a greater similarity at the gene expression level to bacterial cells isolated from infection. Such stresses are likely to be representative of those encountered *in vivo* [44]. It is hypothesized, as a result of this study, that artificial induction of the stringent response with serine hydroxamate can mimic the *in vivo* environment bacteria encountered during the infection process and result in a similar pattern of gene expression.

Interestingly, during active stringent response *F. tularensis* up-regulated an equal number of genes to the number of genes down-regulated. From the most significantly differentially expressed genes in the 1 µg ml^−1^ serine hydroxamate condition, 243 were up-regulated and 156 were down-regulated, which demonstrated that *F. tularensis* undergoes genome-wide gene expression changes as a result of nutrient starvation.

The global gene expression profiles generated in this work will inform the selection of future targets for antimicrobial development. Many hypothetical proteins have also been highlighted whose functions are yet to be elucidated, which could have important roles in bacterial virulence and stress responses.

## Methods

### Bacterial strains, culture conditions and RNA isolation

The prototypic type A virulent strain, *F. tularensis* subsp. *tularensis* SCHU S4 was grown routinely on blood cysteine glucose agar (BCGA) containing 4 % cysteine, 4 % histidine, 5 % glucose and 10 % fresh-filtered defibrinated horse blood. Bacterial growth from a freshly streaked plate was used to inoculate 50 ml cultures CDM without dl-serine. Where required, CDM was supplemented with 1 or 10 µg ml^−1^ serine hydroxamate to induce the stringent response. Cultures were grown aerobically at 37 °C with shaking at 250 r.p.m. for 6 h. At this point OD_600_ was measured and 1 ml of bacterial culture removed, serially diluted and 100 µl spread onto BCGA plates to enumerate bacteria. Following overnight incubation 2 ml bacterial culture were frozen down in RNA Bacteria Protect reagent (Qiagen) at a ratio of 2 : 1 (RNA Protect reagent: culture) for subsequent RNA extraction. Total RNA was extracted according to the Qiagen RNeasy kit instructions, and an on-column DNase digestion was performed. All total RNA extracts were diluted 1/100 and assessed for concentration and quality on the Agilent Bioanalyzer, according to the manufacturer’s instructions for the RNA mRNA pico chip protocol (Agilent Technologies). All work undertaken with *Francisella* strains was performed in a containment level III laboratory in accordance with relevant legislative requirements.

### End point RT-PCR

Titanium One-Step RT-PCR Kit (ClonTech) was used to generate a cDNA template and amplify a target region of the *iglC* gene or the 16S rRNA gene as a reference gene for a control reaction. A typical reaction comprised a mastermix of 5 µl 10× one-step buffer, 1 µl 50X dNTP mix, 0.5 µl recombinant RNase inhibitor (40 units µl^−1^), 25 µl thermostabilizing reagent, 10 µl GC melt, 1 µl oligo(dT) primer and 1 µl 50× titanium *Taq* RT enzyme mix. A typical experimental reaction comprised 43.5 µl of the above mastermix, 2 µl experimental primer mix (45 µM each), 1 µl experimental RNA and 3.5 µl dH_2_O to give a total volume of 50 µl. A positive control reaction comprised 43.5 µl mastermix, 1 µl control mouse β-actin primer mix, 1 µl control mouse liver total RNA and 4.5 µl dH_2_O to give a final volume of 50 µl. A negative control of sterile water was also included. PCRs were run on the following thermal cycling protocol; initial cDNA synthesis 50 °C for 1 h, then denaturation at 94 °C for 5 min, 30 cycles of 94 °C for 30 s, 65 °C for 30 s, 68 °C for 1 min, a final extension of 68 °C for 2 min and then 4 °C on hold. PCR products were analysed by agarose gel electrophoresis.

### Library preparation and sequencing

RNA isolates were prepared in triplicate to provide sufficient biological replicates for RNA-seq. Total RNA was depleted for ribosomal RNA using the Ribo-Zero kit (Epibio) according to the manufacturer’s instructions. mRNA libraries were then prepared using Script-Seq RNA-seq library preparation kit (Epicentre), according to the manufacturer’s instructions. The University of Exeter performed 300 bp, paired end-read RNA sequencing using a single lane on the Illumina HiSeq2500.

### RNA-seq data analysis and statistical determination of differentially expressed genes

Raw images were captured using RTA 1.13.48 (Real Time Analysis), then raw sequence files were de-multiplexed and filtered using CASAVA 1.8.2 (Consensus Assessment of Sequence and Variance), a quality filter designed to remove low-quality reads or sections of reads, as well as any sequences derived from the sequencing adaptors or primers. The quality-filtered FASTQ files were mapped to the *F. tularensis* subsp. *tularensis* SCHU S4 genome (NC_006570.2) with TopHat in local alignment mode. The short read alignments were used as the input for HTSeq, a python framework for working with high-throughput sequencing data [45]. Read counts were generated for each consensus coding sequence (CCS) in the reference sequence in HTSeq. Differentially expressed genes were then identified in each condition using the R package DESeq, by comparing the read counts of each CCS in each serine hydroxamate condition. The DESeq package tests for differential expression through the application of negative binomial distribution and shrinkage estimator for the distribution of variance. Normalized expression levels among the samples were obtained by estimating the total sequencing depths for each sample as the median of the ratios of the samples’ counts to geometric mean across all samples. Genes were identified as being differentially expressed when the DESeq-calculated adjusted *P*-value was less than 0.05 and the change in expression was at least 1.5-fold up or down. Further information about statistical analyses can be found in the DESeq vignette (www.bioconductor.org/packages/devel/bioc/vignettes/DESeq/inst/doc/DESeq.pdf). Heat maps were generated in the software environment R, for statistical computing and graphics generation, using the gplots package. Volcano plots were also generated in R using the VolcanoPlot function.

### Lysate preparation for proteomic analysis

50 ml CDM without serine, supplemented with 0, 1 or 10 µg ml^−1^ serine hydroxamate, was inoculated to OD_600_ 0.1 from a fresh BCGA plate of *F. tularensis* SCHU S4 and incubated at 37 °C with shaking for 6 h. OD_600_ readings were taken to check for growth the following day. 1 ml of culture was then centrifuged at 13 000 r.p.m. (14 100 ***g***) for 1 min to pellet cells. Cells were then resuspended in 1 ml SDS buffer comprising 125 mM Tris pH 6.8, 20 % SDS, 20 % glycerol made up to 1 ml with dH_2_O. SDS cell suspensions were then heated to either 60 °C for 60 min or 100 °C for 10 min then stored at −80 °C. Prior to freezing, 10 % of each cell sample was inoculated into 1 ml CDM and immediately plated onto BGGA plates. After incubation for 1 week at 37 °C, BCGA plates were inspected for bacterial growth and samples deemed non-viable were subsequently subjected to proteomic analysis.

### Sample preparation for proteomics

In total, 5 µl *F. tularensis* lysates were resuspended in 5 µl Tris-SDS-glycerol buffer and protein concentrations measured using a direct detector spectrometer. Volumes equating to 120 mg protein were added to 0.5 µl DTT solution and incubated at 56 °C for 30 mins. Protein digest was carried out using FASP protein digestion kit according to the manufacturer’s instructions. Samples were lyophilized using a vacuum concentrator then cleaned up using the C18 Protea Tip SpinTips Sample Prep Kit (Protea) according to the manufacturer’s instructions. Samples were then reconstituted in 60 µl dH_2_0 +0.1 % formic acid buffer (buffer A). An internal standard for subsequent mass spectrometry was prepared comprising 8 µl of enolase stock and 32 µl of buffer A.

### Mass spectrometry

Samples were separated using a nanoAcquity UPLC system (Waters). For the first dimension separation, 1.0 µl of the prepared protein digest (500 ng on column) containing 100 fmol of the internal standard enolase digest was injected onto a Symmetry C18, 180 µm×20 mm trapping cartridge (Waters). After 5 min washing of the trap column, peptides were separated using a 75 µm ID x 200 mm, 1.7 µm BEH130 C18, column (Waters) using a linear gradient of 5 to 40 % B (buffer A=0.1 % formic acid in water, buffer B=0.1 % formic acid in acetonitrile) over 90 min with a wash to 85 % B at a flow rate of 300 nl min^−1^. All separations were automated and performed online to a Waters G2-S HDMS mass spectrometer operating in MS^e^ mode with ion mobility enabled. Data was acquired from 50 to 2000 *m/z* using alternate low and high collision energy (CE) scans. Low CE was 5V and elevated, ramped from 20 to 40V. The lock mass Glu-fibrinopeptide *m/z*=785.8426 (M+2H)+2 at a concentration of 500 fmol µl^−1^ was infused at 250 nl min^−1^ and acquired every 13 s.

### Database searching

The raw mass spectra were processed using ProteinLynx Global Server Ver 3.0 (Waters, Manchester, UK) and the data processed to generate reduced charge state and de-isotoped precursor and associated product ion mass lists. These mass lists were searched against the *F. tularensis* protein sequence. A maximum of one-missed cleavage was allowed for tryptic digestion and the variable modification was set to contain oxidation of methionine, carboxyamidomethylation of cysteine and hydroxylation of aspartic acid, lysine, asparagine and proline.

## Supplementary Data

877 Supplementary File 1
