# Supplementary material for: Whole genome transcriptomics reveals global effects including up-regulation of Francisella pathogenicity island gene expression during active stringent response in the highly virulent Francisella tularensis subsp. tularensis SCHU S4
Source: Microbiology (Reading). 2017 Nov 8;163(11):1664–79. doi: 10.1099/mic.0.000550 (PMC5845702; doi:10.1099/mic.0.000550)
Supplement: Supplementary File 1 [file mic-163-1664-s001.pdf]

**Gene ID**	**Gene Identifier**	**Gene name**	**Product**	**Log2 Fold Change**	**p-value**	**Functional Category**
GeneID:3191523	FTT_0001	dnaA	chromosome replication initiator DnaA	-1.4168487	0.000192475	Stress response
GeneID:3191533	FTT_0002	dnaN	DNA polymerase III subunit beta	-1.4524627	0.000249023	DNA replication
GeneID:3192082	FTT_0006		MFS superfamily proline/ betaine transporter	2.32486485	2.04E-06	Transport
GeneID:3192542	FTT_0007	aspS	Aspartyl-tRNA synthetase.	-1.5464897	4.66E-05	Translation
GeneID:3192126	FTT_0013 c		lipoprotein	2.09196412	0.000180583	Cellular processes
GeneID:3192132	FTT_0014 c	hypothetical protein		-1.7794476	1.20E-05	Hypothetical and unknown function
GeneID:3192094	FTT_0026 c	hypothetical protein	Similar to Q9HWP5 Probable MFS transporter from Pseudomonas aeruginosa	2.16739442	4.21E-07	Transport
GeneID:3192141	FTT_0028 c	hypothetical protein	Similar to YCEE_ECOLI (P25744) Hypothetical transport protein yce from E. coli	1.98714571	1.48E-06	Transport
GeneID:3191756	FTT_0032	nuoB	NADH dehydrogenase subunit B	-1.9208447	1.38E-06	Energy metabolism
GeneID:3192334	FTT_0033	nuoC	NADH dehydrogenase I	-1.5950906	6.24E-05	Energy metabolism
GeneID:3191807	FTT_0034	nuoD	NADH dehydrogenase subunit D	-1.6167114	4.66E-05	Energy metabolism
GeneID:3192398	FTT_0050	infB	translation initiation factor IF-2	-1.8640941	1.76E-06	Translation
GeneID:3190705	FTT_0054		ATPase	1.54226244	0.000255392	Cellular processes

GeneID:3192108	FTT_0059	atpE	ATP synthase F0F1 subunit C. produces ATP from ADP in the presence of a proton gradient across the membrane; subunit C is part of the membrane proton channel F0	-1.5085336	2.48E-05	Energy metabolism
GeneID:3190708	FTT_0067c		glutaredoxin-like protein	-1.5330869	2.92E-05	Cellular processes
GeneID:3190713	FTT_0068	sodB	superoxide dismutase	-2.1928746	9.26E-09	Stress response
GeneID:3192375	FTT_0071c	gltA	citrate synthase	-2.4475732	2.41E-09	Energy metabolism
GeneID:3192101	FTT_0073	sdhD	succinate dehydrogenase hydrophobic membrane anchor protein	-2.6988291	9.58E-11	Energy metabolism
GeneID:3191590	FTT_0074	sdhA	succinate dehydrogenase catalytic and NAD/flavoprotein subunit	-2.1430908	1.01E-07	Energy metabolism

GeneID:3191270	FTT_0075	sdhB	succinate dehydrogenase iron-sulfur subunit. part of four member succinate dehydrogenase enzyme complex that forms a trimeric complex (trimer of tetramers); SdhA/B are the catalytic subcomplex and can exhibit succinate dehydrogenase activity in the absence of SdhC/D which are the membrane components and form cytochrome b556; SdhC binds ubiquinone; oxidizes succinate to fumarate while reducing ubiquinone to ubiquinol;	-2.1344065	1.04E-07	Energy metabolism
GeneID:3190739	FTT_0077	sucB	dihydrolipoamide succinyltransferase component of 2-oxoglutarate dehydrogenase complex	-1.8494387	3.57E-06	Cellular processes
GeneID:3192423	FTT_0084c	hemN	oxygen-independent coproporphyrinogen III oxidase	1.69861577	2.48E-05	Cofactor and vitamin metabolism
GeneID:3192406	FTT_0087	acnA	aconitate hydratase. Catalyzes the conversion of citrate to isocitrate	-2.2179885	2.09E-08	Carbohydrate metabolism

GeneID:3190741	FTT_0090c	hypothetical protein		1.89712783	4.66E-05	Hypothetical and unknown function
GeneID:3192159	FTT_0096	hypothetical protein		1.90644078	1.37E-06	Hypothetical and unknown function
GeneID:3192163	FTT_0101	hypothetical protein	Similar to Y402_RICPR (Q9ZDC9) Hypothetical protein RP402 from Rickettsia prowazekii	-2.0064272	1.20E-07	Hypothetical and unknown function
GeneID:3190746	FTT_0105c		AcrB/AcrD/AcrF family transporter	1.5891125	0.000137076	Transport
GeneID:3191543	FTT_0107c	dsbB	disulfide bond formation protein	1.65925774	7.13E-05	Transport
GeneID:3191032	FTT_0115	nupC1	nucleoside permease NUP family protein	1.61084772	0.000121184	Transport
GeneID:3191029	FTT_0116	nupC1	nucleoside permease NUP family protein	2.23590163	0.000227251	Transport
GeneID:3191181	FTT_0126	oppF	oligopeptide ABC transporter ATP-binding protein	1.79342654	1.04E-05	Transport
GeneID:3191077	FTT_0132	glpA	anaerobic glycerol-3-phosphate dehydrogenase	-1.5332738	9.56E-05	Energy metabolism
GeneID:3190774	FTT_0136		helicase	1.55071053	7.48E-05	DNA replication

GeneID:3190712	FTT_0137	tufA	elongation factor Tu. promotes GTP-dependent binding of aminoacyl-tRNA to the A-site of ribosomes during protein biosynthesis; when the tRNA anticodon matches the mRNA codon, GTP hydrolysis results; the inactive EF-Tu-GDP leaves the ribosome and release of GDP is promoted by elongation factor Ts; many prokaryotes have two copies of the gene encoding EF-Tu	-1.9298141	2.03E-06	Protein synthesis
GeneID:3191695	FTT_0138	secE	preprotein translocase subunit SecE. forms a complex with SecY and SecG; SecYEG forms a protein-conducting channel to which secA binds and translocates targeted polypeptides across the cytoplasmic membrane, a process driven by ATP and a proton-motive force	-1.311396	0.000295842	Transport

GeneID:3192014	FTT_0140	rplK	50S ribosomal protein L11. binds directly to 23S ribosomal RNA	-1.6021566	1.44E-05	Ribosomal proteins
GeneID:3192023	FTT_0141	rplA	50S ribosomal protein L1	-1.4544063	0.000113437	Ribosomal proteins
GeneID:3192015	FTT_0142	rplJ	50S ribosomal protein L10. binds the two ribosomal protein L7/L12 dimers and anchors them to the large ribosomal subunit	-1.5352011	9.78E-06	Ribosomal proteins
GeneID:3192013	FTT_0143	rplL		-1.8218089	1.37E-06	Ribosomal proteins
GeneID:3192286	FTT_0152	trmD	tRNA (guanine-N(1)-)-methyltransferase. methylates guanosine-37 in various tRNAs; uses S-adenosyl-L-methionine to transfer methyl group to tRNA	-1.254885	0.000263211	DNA replication
GeneID:3192004	FTT_0153	rplS	50S ribosomal protein L19	-1.4063463	6.45E-05	Ribosomal proteins
GeneID:3190874	FTT_0157 c		licB-like transmembrane protein	2.05320989	8.41E-07	Transport
GeneID:3192337	FTT_0158 c	hypothetical protein		2.44436782	1.20E-05	Hypothetical and unknown function
GeneID:3192487	FTT_0162	ampD	N-acetyl-anhydromuranmyl-L-alanine amidase	-2.3690502	4.35E-09	Amino acid biosynthesis
GeneID:3192407	FTT_0164 c		eflux protein	1.552956	0.000167731	Transport
GeneID:3191135	FTT_0169		HemK protein	2.1859083	6.24E-05	Cellular processes

GeneID:3191454	FTT_0174	hypothetical protein	typeII SS	1.76761264	0.00027044	Transport
GeneID:3191366	FTT_0181 c	hypothetical protein	Similar to Q9X885 small integral membrane protein from Streptomyces coelicolor	-1.4825916	0.000134215	Membrane protein
GeneID:3191976	FTT_0183 c	rpsA	30S ribosomal protein S1. involved in binding to the leader sequence of mRNAs and is itself bound to the 30S subunit; autoregulates expression via a C-terminal domain; in most gram negative organisms this protein is composed of 6 repeats of the S1 domain while in gram positive there are 4 repeats; the S1 nucleic acid-binding domain is found associated with other proteins	-2.1113315	4.54E-08	Ribosomal proteins
GeneID:3191833	FTT_0187	ftsA	cell division protein FtsA	-1.8301072	2.22E-06	Cellular processes
GeneID:3191126	FTT_0188	ftsZ	cell division protein FtsZ. GTPase; similar structure to tubulin; forms ring-shaped polymers at the site of cell division	-2.0239219	1.92E-07	Cellular processes

GeneID:3190839	FTT_0189	lpxC	UDP-3-O-[3-hydroxymyristoyl] N-acetylglucosamine deacetylase	-1.6965717	1.70E-05	Glycan Biosynthesis
GeneID:3191362	FTT_0193 c	hypothetical protein		-1.5365167	0.000129337	Hypothetical and unknown function
GeneID:3191892	FTT_0196 c	glnA	glutamine synthetase. Similar to AAO90051 (Q83E31) Glutamine synthetase from Coxiella burnetti	-2.091523	1.96E-07	Amino acid biosynthesis
GeneID:3191352	FTT_0207 c		ABC transporter permease	1.96757922	6.69E-06	Transport
GeneID:3191350	FTT_0209 c		periplasmic solute binding family protein	-1.9478887	7.49E-07	Transport
GeneID:3192410	FTT_0221	acpA	acid phosphatase	-1.4640696	0.000127962	Carbohydrate metabolism
GeneID:3192142	FTT_0222 c	ybgK	hydrolase subunit	1.5346811	0.000137076	Cellular processes
GeneID:3191187	FTT_0228 c	orn	oligoribonuclease	1.94029025	2.92E-05	Cellular processes
GeneID:3191506	FTT_0232 c	ddg	acyltransferase	2.3642306	4.35E-09	Transport
GeneID:3191332	FTT_0244		DNA/RNA helicase	1.90380123	8.04E-07	DNA replication
GeneID:3191912	FTT_0245	usp	universal stress protein	-2.7853607	5.77E-12	Stress response
GeneID:3191325	FTT_0254 c	pseudogene	unknown	-1.6046625	0.000222881	Hypothetical and unknown function
GeneID:3191322	FTT_0256 c		lipopolysaccharide protein	-1.4025512	0.000282262	Cellular processes

GeneID:3191891	FTT_0260	crcB	camphor resistance protein CrcB Putative fluoride ion transporter CrcB [SwissProt]	1.75869298	6.68E-05	Transport
GeneID:3190842	FTT_0271	ipk	4-diphosphocytidyl-2-C-methyl-D-erythritol kinase. An essential enzyme in the nonmevalonate pathway of isopentenyl diphosphate and dimethylallyl diphosphate biosynthesis	1.89433925	7.55E-06	Cellular processes
GeneID:3191907	FTT_0278 c	cydB	cytochrome d terminal oxidase polypeptide subunit II	-1.6146762	1.53E-05	Energy metabolism
GeneID:3191902	FTT_0279 c	cydA	cytochrome d terminal oxidase polypeptide subunit I	-1.7691217	2.13E-06	Carbohydrate metabolism
GeneID:3192388	FTT_0285	cyoE	protoheme IX farnesyltransferase. converts protoheme IX and farnesyl diphosphate to heme O	1.69882061	3.03E-05	Energy metabolism
GeneID:3191297	FTT_0292	hypothetical protein		2.03303882	9.58E-06	Hypothetical and unknown function
GeneID:3191293	FTT_0295	hypothetical protein		1.70004416	1.50E-05	Hypothetical and unknown function
GeneID:3191292	FTT_0297		cytochrome c-type biogenesis protein	-1.6979119	0.000214715	Energy metabolism
GeneID:3191975	FTT_0313	rpsB	30S ribosomal protein S2	-1.5336506	8.79E-05	Ribosomal proteins

GeneID:3191005	FTT_0314	tsf	elongation factor Ts. functions during elongation stage of protein translation; forms a dimer; associates with EF-Tu-GDP complex and promotes exchange of GDP to GTP resulting in regeneration of the active form of EF-Tu	-1.7643204	3.44E-06	Translation
GeneID:3191538	FTT_0321	rpsL	30S ribosomal protein S12. interacts with and stabilizes bases of the 16S rRNA that are involved in tRNA selection in the A site and with the mRNA backbone; located at the interface of the 30S and 50S subunits, it traverses the body of the 30S subunit contacting proteins on the other side; mutations in the S12 gene confer streptomycin resistance	-2.2310474	1.08E-08	Ribosomal proteins

GeneID:3191970	FTT_0322	rpsG	30S ribosomal protein S7. binds directly to 16S rRNA where it nucleates assembly of the head domain of the 30S subunit	-2.2984932	1.04E-07	Ribosomal proteins
GeneID:3191127	FTT_0323	fusA	elongation factor G. promotes GTP-dependent translocation of the ribosome during translation; many organisms have multiple copies of this gene	-2.4312324	1.70E-07	Protein synthesis
GeneID:3191189	FTT_0324	rpsJ	30S ribosomal protein S10	-1.8622144	3.78E-06	Ribosomal proteins
GeneID:3192021	FTT_0325	rplC	50S ribosomal protein L3	-1.9334999	1.30E-06	Ribosomal proteins
GeneID:3192020	FTT_0326	rplD	50S ribosomal protein L4.	-1.610729	1.97E-05	Ribosomal proteins
GeneID:3192000	FTT_0330	rplV	50S ribosomal protein L22. binds specifically to 23S rRNA during the early stages of 50S assembly; makes contact with all 6 domains of the 23S rRNA in the assembled 50S subunit and ribosome; mutations in this gene result in erythromycin resistance; located near peptidyl-transferase center	-1.3880068	0.000175132	Ribosomal proteins

GeneID:3192011	FTT_0335	rplN	50S ribosomal protein L14. binds to the 23S rRNA between the centers for peptidyl transferase and GTPase	-1.7397432	1.83E-06	Ribosomal proteins
GeneID:3192018	FTT_0337	rplE	50S ribosomal protein L5	-1.6240384	4.20E-06	Ribosomal proteins
GeneID:3192379	FTT_0338	rpsN	30S ribosomal protein S14	-1.9723084	1.57E-07	Ribosomal proteins
GeneID:3192017	FTT_0340	rplF	50S ribosomal protein L6	-1.8023182	1.49E-06	Ribosomal proteins
GeneID:3192006	FTT_0341	rplR	50S ribosomal protein L18	-1.8528703	1.98E-06	Ribosomal proteins
GeneID:3191860	FTT_0347	rpsM	30S ribosomal protein S13	-1.4152052	8.30E-05	Ribosomal proteins
GeneID:3190768	FTT_0348	rpsK	30S ribosomal protein S11	-1.6725106	5.47E-06	Ribosomal proteins
GeneID:3191445	FTT_0362c	hypothetical protein		2.09428252	0.000266015	Hypothetical and unknown function
GeneID:3192066	FTT_0368c	mviN	virulence factor MviN. Similar to AAO89943 (Q83ED5) Integral membrane protein MviN from Coxiella burnetii	2.06248482	2.75E-06	Virulence - other
GeneID:3191466	FTT_0372c	accD	acetyl-Coa carboxylase beta subunit	-1.8742691	6.61E-07	Fatty acid biosynthesis
GeneID:3191440	FTT_0376c	hypothetical protein	weak hits at position aa 250-350 to many cytochrome oxidase subunit I	2.32187626	1.58E-07	Hypothetical and unknown function
GeneID:3191438	FTT_0383	hypothetical protein		-2.9395844	2.89E-12	Regulation

GeneID:3191512	FTT_0403	def1	peptide deformylase	-2.341759	9.26E-09	Amino acid biosynthesis
GeneID:3192518	FTT_0407	gcvT	lycine cleavage system aminomethyltransferase T. catalyzes the transfer of a methylene carbon from the methylamine-loaded GcvH protein to tetrahydrofolate, causing the release of ammonia and the generation of reduced GcvH protein	-2.2440525	2.90E-08	Amino acid biosynthesis
GeneID:3191553	FTT_0408	gcvH1	glycine cleavage system H protein	-2.9756632	2.89E-12	Amino acid biosynthesis
GeneID:3191363	FTT_0414	pgm	phosphoglucomutase. catalyzes the interconversion of alpha-D-glucose 1-phosphate to alpha-D-glucose 6-phosphate	-1.7475963	8.42E-06	Carbohydrate metabolism
GeneID:3191414	FTT_0447 c	hypothetical protein		2.00552024	2.03E-06	Hypothetical and unknown function
GeneID:3191267	FTT_0461	yhbY	RNA-binding protein	-1.5315019	7.15E-05	Translation
GeneID:3192548	FTT_0471	aroD	3-dehydroquinate dehydratase. catalyzes the formation of 3-dehydroshikimate from 3-dehydroquinate in chorismate biosynthesis	-2.0576749	1.24E-07	Amino acid biosynthesis
GeneID:3191467	FTT_0472	accB	acetyl-Coa carboxylase biotin carboxyl carrier protein subunit	-2.3931035	1.59E-09	Energy metabolism

GeneID:3192529	FTT_0473	accC	acetyl-Coa carboxylase biotin carboxylase subunit	-1.8997748	1.33E-06	Fatty acid biosynthesis
GeneID:3191405	FTT_0474	hypothetical protein		-2.2145198	1.77E-08	Membrane protein
GeneID:3191637	FTT_0475	msc	mechanosensitive ion channel MscS	1.97799366	1.25E-06	Transport
GeneID:3191874	FTT_0476 c	poxA	lysyl-tRNA synthetase	1.76517534	1.27E-05	Translation
GeneID:3191398	FTT_0487			2.12955021	0.00015678	Hypothetical and unknown function
GeneID:3190740	FTT_0503 c	sucD	succinyl-CoA synthetase subunit alpha	-2.0271695	2.38E-07	Carbohydrate metabolism
GeneID:3191381	FTT_0505	hypothetical protein		-1.8141859	4.20E-06	Hypothetical and unknown function
GeneID:3191384	FTT_0506 c	hypothetical protein		2.26494616	3.07E-08	Hypothetical and unknown function
GeneID:3191213	FTT_0527	pseudogene	unknown	1.69605783	3.51E-05	Hypothetical and unknown function
GeneID:3192520	FTT_0533 c	grxA	glutaredoxin	-1.8217243	5.45E-06	Nucleotide synthesis
GeneID:3191869	FTT_0535 c	mdh	malate dehydrogenase. Catalyzes the reversible oxidation of malate to oxaloacetate	-1.5983592	1.46E-05	Carbohydrate metabolism
GeneID:3192402	FTT_0541 c	yqaB	haloacid dehalogenase	-1.9386992	1.14E-06	Cellular processes
GeneID:3191204	FTT_0542	hypothetical protein		2.36730522	4.28E-08	Hypothetical and unknown function
GeneID:3191202	FTT_0546	hypothetical protein		2.02679785	2.30E-06	Hypothetical and unknown function
GeneID:3191199	FTT_0547	pseudogene	unknown	2.04810806	2.08E-05	Hypothetical and unknown function
GeneID:3191197	FTT_0552		aldehyde dehydrogenase	1.89637906	1.08E-06	Carbohydrate metabolism

GeneID:3190960	FTT_0560 c	serC	phosphoserine aminotransferase. catalyzes the formation of 3-phosphonooxypyruvate and glutamate from O-phospho-L-serine and 2-oxoglutarate; required both in major phosphorylated pathway of serine biosynthesis and in the biosynthesis of pyridoxine	-2.3603366	3.73E-09	Amino acid biosynthesis
GeneID:3191563	FTT_0562	potG	polyamine transporter ABC transporter ATP-binding protein	-1.4568448	0.000183905	Transport
GeneID:3191188	FTT_0569 c	hypothetical protein	Similar to Q8P3T2 Integral membrane protein from Xanthomonas campestris	1.64398962	2.42E-05	Membrane protein
GeneID:3191184	FTT_0572		proton-dependent oligopeptide transport (POT) family protein	1.63288877	0.000147785	Transport
GeneID:3192275	FTT_0577	sdaA	L-serine dehydratase 1	-2.5022843	3.68E-10	Amino acid biosynthesis
GeneID:3192005	FTT_0583	fopA1	outer membrane associated protein	-2.3391931	8.45E-10	Membrane protein
GeneID:3191175	FTT_0586	hypothetical protein	putative RecB family exonuclease	2.11905064	0.000111481	DNA replication

GeneID:3191171	FTT_0594c	hypothetical protein	Similar to Q87N89 Hypothetical protein from Vibrio parahaemolyticus	2.29991914	1.73E-08	Amino acid biosynthesis
GeneID:3191088	FTT_0595c	rubA	rubredoxin	1.71851417	2.81E-05	Hypothetical and unknown function
GeneID:3191169	FTT_0597	hypothetical protein		1.50799906	7.43E-05	Hypothetical and unknown function
GeneID:3191162	FTT_0603	hypothetical protein		2.63371179	3.75E-05	Hypothetical and unknown function
GeneID:3191159	FTT_0604	hypothetical protein		2.34559803	0.000140976	Hypothetical and unknown function
GeneID:3190876	FTT_0607	ispG	4-hydroxy-3-methylbut-2-en-1-yl diphosphate synthase	-2.3158929	4.35E-09	Fatty acid biosynthesis
GeneID:3191155	FTT_0609		peptidase M24 family protein	-1.9527399	8.04E-07	Cellular processes
GeneID:3191151	FTT_0613c	hypothetical protein		-2.4633424	1.59E-09	Hypothetical and unknown function
GeneID:3191149	FTT_0614c		apolipoprotein N-acyltransferase	2.15114801	1.36E-07	Hypothetical and unknown function
GeneID:3191146	FTT_0619		o-methyltransferase family protein	1.99333241	1.36E-05	Transport
GeneID:3191144	FTT_0620	acpC	HAD superfamily protein	2.13211008	8.19E-08	Amino acid biosynthesis

GeneID:3192361	FTT_0624	clpP	ATP-dependent Clp protease proteolytic subunit. hydrolyzes proteins to small peptides; with the ATPase subunits ClpA or ClpX, ClpP degrades specific substrates	-1.4355668	5.48E-05	Stress response
GeneID:3191898	FTT_0627	hupB	histone-like protein HU form B	-2.3189076	3.73E-09	Regulation
GeneID:3192428	FTT_0630	hfq	host factor I for bacteriophage Q beta replication	-2.1788179	2.12E-08	Regulation
GeneID:3192427	FTT_0631	hflX	protease GTP-binding subunit	1.99084892	7.98E-05	Energy metabolism
GeneID:3191139	FTT_0638	hypothetical protein	fragment. Weak similarity to leuA LE12_METJA (Q58595) 2-isopropylmalate synthase 2 from Methanococcus jannaschii	2.01578896	2.06E-05	Hypothetical and unknown function
GeneID:3191138	FTT_0639	hypothetical protein	Weak similarity to leuA LE12_METJA (Q58595) 2-isopropylmalate synthase 2 from Methanococcus jannaschii	1.8475751	0.000248342	Hypothetical and unknown function
GeneID:3191066	FTT_0645c	hypothetical protein		-1.6366883	5.37E-06	Cellular processes
GeneID:3191064	FTT_0647c	hypothetical protein		1.72743526	0.000199302	Hypothetical and unknown function

GeneID:3192277	FTT_0650c	grxB	glutaredoxin. cofactor involved in the reduction of disulfides	-1.6531466	1.18E-05	Cellular processes
GeneID:3191059	FTT_0659		DNA recombination protein RmuC family protein. Similar to Q87TH5 Conserved hypothetical protein from Vibrio parahaemolyticus	1.49587156	0.000110806	DNA replication
GeneID:3191047	FTT_0670c	hypothetical protein		-2.1675197	4.99E-08	Hypothetical and unknown function
GeneID:3191045	FTT_0671		major facilitator transporter	1.49946676	0.000127962	Transport
GeneID:3191230	FTT_0674	prsA	ribose-phosphate pyrophosphokinase	-1.5644079	4.75E-05	Carbohydrate metabolism
GeneID:3190964	FTT_0681c	blaA	beta-lactamase class A	1.50139905	0.000123049	Cellular processes
GeneID:3191481	FTT_0683c	pilD	Type IV pili leader peptidase and methylase	2.34910818	1.96E-07	Virulence - other
GeneID:3191158	FTT_0698	rpsO	30S ribosomal protein S15. primary rRNA binding protein; helps nucleate assembly of 30S; binds directly to the 16S rRNA and an intersubunit bridge to the 23S rRNA; autoregulates translation through interactions with the mRNA leader sequence	-2.1921665	8.17E-09	Ribosomal proteins

GeneID:3191977	FTT_0703	rpoZ	DNA-directed RNA polymerase subunit omega	-1.7604673	2.84E-06	Regulation
GeneID:3191026	FTT_0704	hypothetical protein		-1.519737	2.65E-05	Hypothetical and unknown function
GeneID:3191024	FTT_0708		major facilitator transporter. Similar to Q83BD8 Major facilitator family transporter from Coxiella burnetii	-2.2492516	1.98E-08	Virulence - other
GeneID:3192112	FTT_0714 c	kbl	2-amino-3-ketobutyrate CoA ligase	-1.9554007	7.04E-07	Amino acid biosynthesis
GeneID:3191905	FTT_0716	upp	uracil phosphoribosyltransferase	-1.4423264	0.000192475	Nucleotide synthesis
GeneID:3191021	FTT_0718	hypothetical protein		2.14418288	4.52E-05	Hypothetical and unknown function
GeneID:3191018	FTT_0719		major facilitator transporter	1.77458013	1.04E-05	Transport
GeneID:3191520	FTT_0720 c	dgt	deoxyguanosinetriphosphate triphosphohydrolase.	-2.110768	9.40E-08	Amino acid biosynthesis
GeneID:3192098	FTT_0721 c	katG	peroxidase/catalase	-1.6695789	1.31E-05	Stress response
GeneID:3192154	FTT_0728	ybhF	ABC transporter ATP-binding protein	2.40435442	7.25E-09	Transport
GeneID:3192155	FTT_0729	ybhR	ABC transporter permease	2.47563758	8.04E-06	Transport
GeneID:3192180	FTT_0736	hypothetical protein		2.03851654	6.05E-05	Hypothetical and unknown function
GeneID:3191856	FTT_0744 c	pseudogene	unknown	2.08572898	4.52E-05	Hypothetical and unknown function

GeneID:3191851	FTT_0748	hypothetical protein		-1.8769887	1.33E-06	Hypothetical and unknown function
GeneID:3191517	FTT_0766	deoD	purine nucleoside phosphorylase	-1.7568863	1.18E-06	Nucleotide synthesis
GeneID:3191611	FTT_0776 c	rnd	ribonuclease D	1.52448999	0.000189992	DNA replication
GeneID:3191864	FTT_0782	fabI	enoyl-ACP reductase	-2.1937065	1.06E-08	Fatty acid biosynthesis
GeneID:3190951	FTT_0783	ars	arylsulfatase	2.49616415	0.000101587	Fatty acid biosynthesis
GeneID:3191824	FTT_0784	hypothetical protein		2.20002814	5.37E-07	Hypothetical and unknown function
GeneID:3191822	FTT_0785	hypothetical protein		2.42211517	1.20E-05	Cellular processes
GeneID:3191812	FTT_0798		glycosyl transferase family protein	1.78699824	1.66E-05	Transport
GeneID:3191811	FTT_0799		glycosyl transferases group 1 family protein	2.24726095	7.61E-06	Transport
GeneID:3191810	FTT_0800		haloacid dehalogenase	2.12403511	2.71E-07	Cellular processes
GeneID:3191887	FTT_0802	cphB	cyanophycinase	-1.740177	8.44E-06	Cellular processes
GeneID:3191806	FTT_0814 c	pseudogene	unknown	1.65337539	7.26E-05	Hypothetical and unknown function
GeneID:3191797	FTT_0822	isftu1	transposase	-1.5965057	0.000134215	Mobile element
GeneID:3191835	FTT_0823		MutT protein	-1.3912798	0.000137076	Cellular processes
GeneID:3191761	FTT_0825 c	hypothetical lipoprotein	hypothetical lipoprotein, no significant homologs.	-2.5135636	4.65E-10	Hypothetical and unknown function
GeneID:3191754	FTT_0829 c		aspartate:alanine antiporter	1.48366767	0.000101587	Transport

GeneID:3191752	FTT_0831 c		OmpA family protein. Weakly similar to Q8A8H3 Outer membrane protein,OmpA family from Bacteroides thetaiotaomicron	-2.2600875	1.70E-08	Membrane protein
GeneID:3190808	FTT_0833	ispH	4-hydroxy-3-methylbut-2-enyl diphosphate reductase	-1.8547478	3.23E-06	Cellular processes
GeneID:3190788	FTT_0840	tolB	TolB protein precursor	1.9576029	9.56E-05	Transport
GeneID:3191742	FTT_0846		deoxyribodipyrimidine photolyase	1.8363942	3.57E-06	DNA replication
GeneID:3191738	FTT_0847	hypothetical protein		2.05965076	5.37E-07	Nucleotide synthesis
GeneID:3191725	FTT_0863 c		LemA-like protein	-1.4059779	0.000259449	Hypothetical and unknown function
GeneID:3191721	FTT_0868 c		arsenical restistance operon repressor	1.96753406	5.47E-06	Cellular processes
GeneID:3191715	FTT_0874 c	hypothetical protein		-1.4439859	0.000249346	Hypothetical and unknown function
GeneID:3190715	FTT_0879	sodC	superoxide dismutase (Cu-Zn) precusor. Identical to SODC_FRATU from F. tularensis LVS.	-2.5803689	3.70E-10	Stress response
GeneID:3191712	FTT_0881 c		amino acid permease	-1.4870068	0.000187117	Transport

GeneID:3190956	FTT_0884 c	aspC1	aspartate aminotransferase. catalyzes the formation of oxalozcetate and L-glutamate from L-aspartate and 2-oxoglutarate	-1.9161828	1.10E-06	Amino acid biosynthesis
GeneID:3191707	FTT_0885		cation transporter	2.21981718	1.96E-07	Transport
GeneID:3191705	FTT_0887 c	hypothetical protein		1.53511017	0.000215294	Hypothetical and unknown function
GeneID:3191700	FTT_0890 c		Type IV pili fiber building block protein	-2.7757906	1.69E-11	Virulence - other
GeneID:3191675	FTT_0896	purE	phosphoribosylaminoimidazole carboxylase catalyic subunit	1.51622174	0.000195953	Nucleotide synthesis
GeneID:3191697	FTT_0900	hypothetical protein		-2.3479211	3.15E-08	Membrane protein
GeneID:3191792	FTT_0901	lpnA	lipoprotein	-2.3971768	7.97E-10	Fatty acid biosynthesis
GeneID:3192543	FTT_0915 c	ileS	isoleucyl-tRNA synthetase.	-1.745637	1.15E-05	Translation
GeneID:3192386	FTT_0917	maeA	malate dehydrogenase	-2.0299654	1.72E-07	Carbohydrate metabolism
GeneID:3191684	FTT_0918	hypothetical protein		-2.0546408	1.92E-07	Hypothetical and unknown function
GeneID:3191682	FTT_0919	hypothetical protein		2.41842288	0.000112552	Hypothetical and unknown function
GeneID:3190914	FTT_0920	isftu1	transposase. ISFtu1. Transposase, member of the IS630 Tc-1 mariner family.	2.24549728	0.000137964	Mobile element

GeneID:3192459	FTT_0931		major facilitator transporter	1.97095466	7.65E-07	Transport
GeneID:3190968	FTT_0937c	bioB	biotin synthase	-1.4982325	5.71E-05	Cofactor and vitamin metabolism
GeneID:3192441	FTT_0962		ThiJ/PfpI family protein	1.75852803	1.77E-05	Amino acid biosynthesis
GeneID:3190950	FTT_0963c	aroG	phospho-2-dehydro-3-deoxyheptonate aldolase. catalyzes the formation of 3-deoxy-D-arabino-hept-2-ulosonate 7 phosphate from phosphoenolpyruvate and D-erythrose 4-phosphate, phenylalanine sensitive	-1.5942199	3.82E-05	Amino acid biosynthesis
GeneID:3191883	FTT_0964c	ffh	signal recognition particle protein, Ffh	-1.5997625	3.09E-05	Stress response
GeneID:3190899	FTT_0979c		amino-acid permease	1.57231736	9.93E-05	Cellular processes
GeneID:3190897	FTT_0980	hypothetical protein	Similar to Q8UKI4 Aminotransferase, class II from Agrobacterium tumefaciens	-2.2554966	1.98E-08	Amino acid biosynthesis
GeneID:3190896	FTT_0981	hypothetical protein		-2.8581412	4.79E-12	Hypothetical and unknown function
GeneID:3190892	FTT_0986	hypothetical protein		1.60257936	0.000244194	Hypothetical and unknown function
GeneID:3190889	FTT_0988	hypothetical protein		1.76732709	0.000199164	Hypothetical and unknown function

GeneID:3190887	FTT_0992	hypothetical protein	Similar to Q8A7A2 membrane protein from Bacteroides thetaiotaomicron	2.20952076	8.69E-08	Transport
GeneID:3192069	FTT_1001		regulatory protein. Similar to Q8ZJ15 regulatory protein from Yersinia pestis	-1.7495033	1.16E-05	Regulation
GeneID:3192068	FTT_1006	hypothetical protein		2.20169091	1.36E-07	Transport
GeneID:3192067	FTT_1007 c	pseudogene	unknown	2.36804151	1.48E-06	Hypothetical and unknown function
GeneID:3192056	FTT_1015	hypothetical protein		-2.1696505	2.52E-08	Hypothetical and unknown function
GeneID:3192053	FTT_1020 c		amino acid permease	1.85855445	1.89E-06	Hypothetical and unknown function
GeneID:3191499	FTT_1029	dacD	D-alanyl-D-alanine carboxypeptidase	-1.5592768	5.30E-05	Glycan Biosynthesis
GeneID:3191685	FTT_1034 c	ndh	NADH dehydrogenase	-1.4818424	8.07E-05	Energy metabolism
GeneID:3192031	FTT_1055 c	hypothetical protein		1.65400391	0.000101587	Hypothetical and unknown function
GeneID:3192016	FTT_1060 c	rplI	50S ribosomal protein L9	-1.8548232	2.05E-07	Ribosomal proteins
GeneID:3192527	FTT_1061 c	rpsR	30S ribosomal protein S18. binds as a heterodimer with protein S6 to the central domain of the 16S rRNA; helps stabilize the platform of the 30S subunit	-1.9925811	9.59E-08	Ribosomal proteins

GeneID:3191971	FTT_1062c	rpsF	30S ribosomal protein S6	-1.96096	1.96E-07	Ribosomal proteins
GeneID:3191073	FTT_1066c	hypothetical protein		1.94326987	0.000264296	Hypothetical and unknown function
GeneID:3192288	FTT_1069c	hypothetical protein		1.92703879	1.34E-05	Hypothetical and unknown function
GeneID:3190794	FTT_1071c	hypothetical protein		2.44282514	7.87E-06	Hypothetical and unknown function
GeneID:3191964	FTT_1073c	pseudogene	unknown	2.15087139	8.13E-06	Hypothetical and unknown function
GeneID:3191631	FTT_1087c	rep	ATP-dependent DNA helicase	1.87361473	7.34E-05	DNA replication
GeneID:3191951	FTT_1089	hypothetical protein	Hypothetical protein from Mycoplasma penetrans	-2.2734165	1.07E-08	Hypothetical and unknown function
GeneID:3190749	FTT_1093c	talA	transaldolase B	-1.625035	1.83E-05	Carbohydrate metabolism
GeneID:3191250	FTT_1103	(mip/dsbA)	lipoprotein. Similar to Q8Z9P6 exported protein from Salmonella typhi	-2.0779725	7.33E-08	Virulence - other
GeneID:3191247	FTT_1110	hypothetical protein		-1.8963541	8.04E-07	Hypothetical and unknown function

GeneID:3191076	FTT_1114 c	secF	preprotein translocase subunit SecF. forms a complex with SecD and YajC; SecDFyajC stimulates the proton motive force-driven protein translocation; seems to modulate the cycling of SecA by stabilizing its membrane-inserted state and appears to be required for the release of mature proteins from the extracytoplasmic side of the membrane	2.11457398	6.20E-07	Transport
GeneID:3191719	FTT_1115 c	secD	preprotein translocase subunit SecD	1.37544286	0.000294106	Transport
GeneID:3191233	FTT_1118 c	hypothetical protein		1.62518293	0.00012872	Hypothetical and unknown function
GeneID:3190821	FTT_1124	metN	D-methionine ABC transporter ATP-binding protein	-1.6086467	6.71E-05	Transport
GeneID:3192171	FTT_1127		rhodanese-like family protein	-1.5016851	0.000118243	Cellular processes
GeneID:3191730	FTT_1129 c	hypothetical protein		-1.5876284	7.16E-05	Hypothetical and unknown function

GeneID:3191888	FTT_1130 c	cphA	cyanophycin synthetase. catalyze the formation of cyanophycin which may act to store excess nitrogen	-1.618321	4.89E-05	Cellular processes
GeneID:3191658	FTT_1136 c	hypothetical protein		-1.3729355	0.000197172	Cellular processes
GeneID:3191969	FTT_1140	hypothetical protein		-2.8535147	4.79E-12	Hypothetical and unknown function
GeneID:3191519	FTT_1147 c	dfp	4'-phosphopantothenoylcysteine decarboxylase	-1.523108	0.000135728	Cofactor and vitamin metabolism
GeneID:3191660	FTT_1150 c	putA	bifunctional proline dehydrogenase/pyrroline-5-carboxylate dehydrogenase	-1.5688756	7.44E-05	Amino acid biosynthesis
GeneID:3192179	FTT_1152	hypothetical protein		-1.6140426	1.61E-05	Hypothetical and unknown function
GeneID:3192541	FTT_1155 c	aroK	shikimate kinase I	-1.3060435	0.000211285	Amino acid biosynthesis
GeneID:3191034	FTT_1158 c		Type IV pili glycosylation protein. Type II SS	1.53727907	0.000230302	Transport
GeneID:3192396	FTT_1161	adk	adenylate kinase. essential enzyme that recycles AMP in active cells; converts ATP and AMP to two molecules of ADP	-1.9243556	1.01E-06	Nucleotide synthesis

GeneID:3190753	FTT_1171c	hsdM	DNA-methyltransferase, type I restriction-modification enzyme subunit M	1.94855445	7.70E-07	Transport
GeneID:3192331	FTT_1177c	pseudogene	unknown	1.61603131	6.22E-05	Hypothetical and unknown function
GeneID:3191007	FTT_1183c		lipoprotein	1.91169902	1.33E-06	Transcription
GeneID:3190989	FTT_1191		aminoacylase	1.9578248	0.000187117	Cellular processes
GeneID:3192282	FTT_1194c		lipoprotein	1.59339604	3.07E-05	Fatty acid biosynthesis
GeneID:3191920	FTT_1199c	uvrB	excinuclease ABC subunit B. The UvrABC repair system catalyzes the recognition and processing of DNA lesions. The beta-hairpin of the Uvr-B subunit is inserted between the strands, where it probes for the presence of a lesion	1.39652269	0.000253239	Transport
GeneID:3192517	FTT_1205	gidA	tRNA uridine 5-carboxymethylaminomethyl modification protein GidA	-1.5272514	9.67E-05	Translation
GeneID:3192232	FTT_1220		5-formyltetrahydrofolate cyclo-ligase	-2.235575	2.09E-08	Cofactor and vitamin metabolism
GeneID:3192229	FTT_1221	hypothetical protein	BolA protein	-1.8558839	2.21E-06	Cellular processes
GeneID:3191509	FTT_1222	dedA2	DedA family protein	-1.5621066	0.000118243	Membrane protein

GeneID:3191610	FTT_1227	rne	ribonuclease E	-1.8894695	1.06E-06	Cellular processes
GeneID:3192211	FTT_1238c	hypothetical protein		1.49810265	0.000241705	Hypothetical and unknown function
GeneID:3191778	FTT_1241	glyA	serine hydroxymethyltransferase. catalyzes the reaction of glycine with 5,10-methylenetetrahydrofolate to form L-serine and tetrahydrofolate	-2.2695227	1.40E-08	Amino acid biosynthesis
GeneID:3192555	FTT_1252	rsmE	16S ribosomal RNA methyltransferase RsmE	-1.6423547	3.07E-05	Cellular processes
GeneID:3191476	FTT_1253		proton-dependent oligopeptide transport (POT) family protein	-2.5054842	4.65E-10	Amino acid biosynthesis
GeneID:3192012	FTT_1273	rplM	50S ribosomal protein L13	-1.5718326	2.05E-05	Ribosomal proteins
GeneID:3191268	FTT_1281c	yhbH	sigma-54 modulation protein	-1.6742119	3.23E-06	Regulation
GeneID:3191096	FTT_1293c		Sua5_yciO_yrdC family protein	-1.6892339	1.84E-05	Hypothetical and unknown function
GeneID:3192511	FTT_1295c	glk1	glucose kinase	-1.4895963	0.000131582	Carbohydrate metabolism
GeneID:3191324	FTT_1318c	pepA	cytosol aminopeptidase	-2.5078602	4.65E-10	Amino acid biosynthesis
GeneID:3191117	FTT_1322		peptidase M16 family protein	1.41116272	0.000268939	Amino acid biosynthesis

GeneID:3190945	FTT_1329	gpmI	phosphoglyceromutase. catalyzes the interconversion of 2-phosphoglycerate and 3-phosphoglycerate	-2.0252893	2.72E-07	Carbohydrate metabolism
GeneID:3190714	FTT_1330	serS	seryl-tRNA synthetase. catalyzes a two-step reaction, first charging a serine molecule by linking its carboxyl group to the alpha-phosphate of ATP, followed by transfer of the aminoacyl-adenylate to its tRNA	-2.0547103	1.51E-07	Hypothetical and unknown function
GeneID:3191125	FTT_1333c	hypothetical protein		-2.5458134	7.17E-09	Hypothetical and unknown function
GeneID:3191128	FTT_1334c	hypothetical protein		-2.7268935	2.35E-11	Hypothetical and unknown function
GeneID:3191294	FTT_1344	pdpA1	hypothetical protein (TSSS)	-2.5021423	7.40E-10	Virulence - FPI
GeneID:3191301	FTT_1345	pdpB1	hypothetical protein (TSSS)	-1.8859421	2.13E-06	Virulence - FPI
GeneID:3191570	FTT_1346	hypothetical protein		-2.0837488	1.72E-05	Hypothetical and unknown function
GeneID:3191577	FTT_1347	hypothetical protein	Similar to AAP58969.1 (Q7XI38) unknown protein from Francisella novicida	-2.4303835	1.73E-08	Amino acid biosynthesis
GeneID:3191580	FTT_1349	hypothetical protein		-2.1892375	5.87E-08	Hypothetical and unknown function

GeneID:3191581	FTT_1350	hypothetical protein	Similar to AAP58973.1 (Q7X3I4) unknown protein from Francisella novicida	-2.1117288	1.40E-07	Hypothetical and unknown function
GeneID:3191586	FTT_1351	hypothetical protein	Similar to AAP58974.1 (Q7X3I3) unknown protein from Francisella novicida	-2.3332878	2.09E-08	Hypothetical and unknown function
GeneID:3191588	FTT_1352	hypothetical protein		-1.6350895	6.24E-05	Hypothetical and unknown function
GeneID:3191518	FTT_1356 c	iglD1	intracellular growth locus subunit D	-2.5943833	2.75E-09	Virulence - FPI
GeneID:3191591	FTT_1357 c	iglC1	intracellular growth locus subunit C	-2.7013555	5.03E-11	Virulence - FPI
GeneID:3191564	FTT_1358 c	iglB1	intracellular growth locus subunit B	-3.0033874	2.97E-11	Virulence - FPI
GeneID:3191217	FTT_1359 c	iglA1	intracellular growth locus subunit A	-2.6353738	1.44E-10	Virulence - FPI
GeneID:3191310	FTT_1360 c	pdpD1	hypothetical protein (TSSS)	-2.5889407	1.96E-10	Virulence - FPI
GeneID:3191657	FTT_1366 c	pyk	pyruvate kinase	-1.5557759	0.000151864	Amino acid biosynthesis
GeneID:3191360	FTT_1367 c	pgk	phosphoglycerate kinase. Converts 3-phospho-D-glycerate to 3-phospho-D-glyceroyl phosphate during the glycolysis pathway	-2.2695706	3.16E-08	Energy metabolism
GeneID:3191592	FTT_1368 c	gapA	glyceraldehyde-3-phosphate dehydrogenase	-2.3657729	5.38E-09	Energy metabolism

GeneID:3191598	FTT_1370	hypothetical protein		-2.0166015	2.05E-07	Hypothetical and unknown function
GeneID:3192405	FTT_1376	acpP	acyl carrier protein	-1.8981838	2.20E-07	Fatty acid biosynthesis
GeneID:3191485	FTT_1384 c		acetyltransferase	-1.853567	1.50E-06	Hypothetical and unknown function
GeneID:3191491	FTT_1388	hypothetical protein		-2.7714619	1.69E-11	Hypothetical and unknown function
GeneID:3191227	FTT_1389	panB	3-methyl-2-oxobutanoate hydroxymethyltransferase. catalyzes the formation of tetrahydrofolate and 2-dehydropantoate from 5,10-methylenetetrahydrofolate and 3-methyl-2-oxobutanoate	-2.1556801	1.50E-07	Hypothetical and unknown function
GeneID:3191378	FTT_1390	panC	pantoate-beta-alanine ligase	-1.7990482	6.14E-06	Amino acid biosynthesis
GeneID:3191493	FTT_1392		pantothenate kinase	-1.8643631	2.59E-06	Cofactor and vitamin metabolism
GeneID:3190879	FTT_1400 c	hypothetical protein		-1.7349286	1.16E-06	Cellular processes
GeneID:3191880	FTT_1403 c	feoA	ferrous iron transport protein A	-1.445932	0.000154717	Transport
GeneID:3192493	FTT_1424 c	hypothetical protein		-1.5135305	0.000127962	Hypothetical and unknown function
GeneID:3190918	FTT_1427	isftu1	transposase. ISFtu1. Transposase, member of the IS630 Tc-1 mariner family.	2.21843989	0.00014063	Mobile element

GeneID:3192325	FTT_1428 c		acetyltransferase	1.54342718	0.000111671	Amino acid biosynthesis
GeneID:3191627	FTT_1431	rhtC	threonine efflux protein	1.57611164	0.000211375	Transport
GeneID:3190796	FTT_1441	hypothetical protein		-1.8247126	4.48E-07	Cellular processes
GeneID:3191983	FTT_1442 c		DNA-directed RNA polymerase subunit alpha	-1.5386365	1.07E-05	Transcription
GeneID:3192113	FTT_1453 c	wzx	O-antigen flippase	2.07470496	1.26E-07	Virulence - other
GeneID:3192088	FTT_1454 c	wbtJ	hypothetical protein	-1.6076407	2.00E-05	Hypothetical and unknown function
GeneID:3192114	FTT_1458 c	wzy	membrane protein/O-antigen protein. membrane protein/O-antigen polymerase	1.7093711	4.89E-05	Membrane protein
GeneID:3192075	FTT_1467 c	nadB	L-aspartate oxidase	1.73315108	1.31E-05	Amino acid biosynthesis
GeneID:3192184	FTT_1472 c	ppiC	peptidyl-prolyl cis-trans isomerase	-1.5689934	6.64E-06	Virulence - other
GeneID:3192412	FTT_1484 c	aceF	dihydrolipoamide acetyltransferase	-1.9079515	2.30E-06	Carbohydrate metabolism
GeneID:3192411	FTT_1485 c	aceE	pyruvate dehydrogenase subunit E1	-1.9879849	5.87E-07	Carbohydrate metabolism
GeneID:3190829	FTT_1486 c	hypothetical protein		1.69300695	0.000119062	Hypothetical and unknown function
GeneID:3192492	FTT_1490		Na+/H+ antiporter	1.82721133	4.75E-06	Transport
GeneID:3190800	FTT_1491 c	pseudogene	unknown	2.20200388	4.62E-05	Hypothetical and unknown function
GeneID:3192314	FTT_1495 c	hypothetical protein		1.43395529	0.000137076	Hypothetical and unknown function

GeneID:3192200	FTT_1498 c	accA	acetyl-Coa carboxylase carboxyltransferase subunit alpha. catalyzes the carboxylation of acetyl-CoA to malonyl-CoA; forms a tetramer composed of two alpha (AccA) and two beta (AccD) subunits; one of the two catalytic subunits that can form the acetyl CoA carboxylase enzyme together with a carrier protein	-2.0755782	9.30E-08	Energy metabolism
GeneID:3190870	FTT_1506	hypothetical protein		-1.6438744	3.11E-05	Hypothetical and unknown function
GeneID:3191763	FTT_1510 c		aromatic amino acid HAAP transporter	1.43169838	0.000263141	Transport
GeneID:3191571	FTT_1520 c	gabP	glutamate/gamma-aminobutyrate anti-porter	2.37725135	1.38E-06	Transport
GeneID:3191012	FTT_1522 c	hypothetical protein		1.85484046	8.36E-06	Hypothetical and unknown function
GeneID:3192408	FTT_1524 c	hrpA	ATp-dependent helicase	1.95777555	5.89E-06	DNA replication
GeneID:3190818	FTT_1525 c	hypothetical protein		-2.1323456	4.50E-08	Hypothetical and unknown function
GeneID:3192248	FTT_1526 c	idh	Isocitrate dehydrogenase,NADP-dependent	-2.2787592	1.07E-08	Energy metabolism

GeneID:3191875	FTT_1528	fadD2	long chain fatty acid CoA ligase	-2.0753385	1.86E-07	Fatty acid biosynthesis
GeneID:3191873	FTT_1529	fadE	acyl-CoA dehydrogenase. functions in fatty acid oxidation; converts acyl-CoA and FAD to FADH2 and delta2-enoyl-CoA	-2.2798442	1.92E-07	Fatty acid biosynthesis
GeneID:3191871	FTT_1530	fadB/acbP	bifunctional 3-hydroxacyl-CoA dehydrogenase/acyl-CoA-binding protein	-2.2503072	2.61E-08	Fatty acid biosynthesis
GeneID:3191866	FTT_1531	fadA	acetyl-CoA acetyltransferase. Catalyzes the synthesis of acetoacetyl coenzyme A from two molecules of acetyl coenzyme A. It can also act as a thiolase, catalyzing the reverse reaction and generating two-carbon units from the four-carbon product of fatty acid oxidation	-2.3554264	6.99E-08	Energy metabolism
GeneID:3192292	FTT_1536c	hypothetical protein		-1.7068072	2.00E-05	Hypothetical and unknown function
GeneID:3190869	FTT_1539c	hypothetical protein	Similar to Q9PJ34 periplasmic protein from Campylobacter jejuni	-2.1048831	9.75E-08	Hypothetical and unknown function
GeneID:3192320	FTT_1540c	hypothetical protein		-1.530579	9.66E-05	Hypothetical and unknown function

GeneID:3191140	FTT_1542 c	omp26	hypothetical protein Similar to Q57483 Outer membrane protein 26	-2.5590277	1.44E-10	Membrane protein
GeneID:3192255	FTT_1549	hypothetical protein		2.4217736	2.75E-06	Hypothetical and unknown function
GeneID:3192230	FTT_1550	hypothetical protein		1.97872628	2.59E-05	Hypothetical and unknown function
GeneID:3190994	FTT_1554 c	truB	tRNA pseudouridine synthase B	1.46123645	0.000166792	Cellular processes
GeneID:3191612	FTT_1555 c	rnc	ribonuclease III. cytoplasmic enzyme involved in processing rRNA and some mRNAs; substrates typically have dsRNA regions; forms a homodimer; have N-terminal nuclease and C-terminal RNA-binding domains; requires magnesium as preferred ion for activity	1.95602404	3.11E-06	DNA replication
GeneID:3190787	FTT_1557 c		two-component response regulator. Similar to AAO90736 DNA-binding response regulator from Coxiella burnetii	-1.418576	5.10E-05	Stress response
GeneID:3191290	FTT_1563	pcs	phosphatidylcholine synthase	1.50461902	0.000266015	Fatty acid biosynthesis

GeneID:3190814	FTT_1569 c	lpxA	UDP-N-acetylglucosamine acyltransferase. catalyzes the addition of (R)-3-hydroxytetradecanoyl to the glucosamine disaccharide in lipid A biosynthesis	-1.4748589	6.81E-05	Glycan Biosynthesis
GeneID:3191868	FTT_1570 c	fabZ	(3R)-hydroxymyristoyl-ACP dehydratase	-1.6586213	5.47E-06	Fatty acid biosynthesis
GeneID:3190807	FTT_1571 c	lpxD	UDP-3-O-[3-hydroxymyristoyl] glucosamine N-acyltransferase	-1.3952937	0.000104074	Glycan Biosynthesis
GeneID:3191143	FTT_1572 c	ompH	outer membrane protein OmpH	-1.4453477	3.10E-05	Membrane protein
GeneID:3192167	FTT_1573 c	hypothetical protein	Similar to Q88MH2 Outer membrane protein,bacterial surface antigen family from Pseudomonas putida	2.05721177	1.08E-07	Membrane protein
GeneID:3192137	FTT_1576	hypothetical protein		2.30097249	1.37E-07	Hypothetical and unknown function
GeneID:3192144	FTT_1577	hypothetical protein		2.10729451	4.87E-05	Hypothetical and unknown function
GeneID:3192096	FTT_1579 c		Type III restriction enzyme	1.83526899	2.44E-06	DNA replication
GeneID:3191930	FTT_1586 c	hypothetical protein		-2.8348356	5.03E-11	Hypothetical and unknown function

GeneID:3191501	FTT_1597	hypothetical protein		2.15830789	1.96E-07	Hypothetical and unknown function
GeneID:3191534	FTT_1600c	fumA	fumerate hydratase	-1.7364901	7.91E-06	Carbohydrate metabolism
GeneID:3192434	FTT_1606	minD	septum site-determining protein MinD	-1.7321175	1.76E-06	Cellular processes
GeneID:3191486	FTT_1608		ABC transporter ATP-binding protein	1.57923959	6.26E-05	Transport
GeneID:3191579	FTT_1609		ABC transporter permease	1.45368593	0.000266015	Transport
GeneID:3192192	FTT_1626c	hypothetical protein		2.16077029	6.52E-08	Hypothetical and unknown function
GeneID:3191084	FTT_1627c	hypothetical protein		1.98350244	0.000206404	Hypothetical and unknown function
GeneID:3192479	FTT_1629c	hypothetical protein		2.34365545	8.71E-08	Transport
GeneID:3190827	FTT_1639c	hypothetical protein		-2.2032024	2.05E-08	Hypothetical and unknown function
GeneID:3192336	FTT_1644		beta-fructofuranosidase	2.08796879	3.17E-06	Carbohydrate metabolism
GeneID:3191089	FTT_1645	hypothetical protein		2.32885242	1.09E-05	Hypothetical and unknown function
GeneID:3190851	FTT_1650c		chorismate mutase	-1.9263033	1.38E-06	Hypothetical and unknown function
GeneID:3190846	FTT_1653	hypothetical protein		-1.7342769	1.52E-06	Hypothetical and unknown function
GeneID:3192365	FTT_1666c		3-hydroxyisobutyrate dehydrogenase	-2.8878414	4.69E-12	Amino acid biosynthesis
GeneID:3191410	FTT_1668	sdaC2	serine transporter	2.1792503	7.52E-07	Transport
GeneID:3192344	FTT_1685	pseudogene	unknown	2.21874905	1.48E-06	Hypothetical and unknown function
GeneID:3192349	FTT_1688		aromatic amino acid HAAP transporter	1.68406589	2.54E-05	Transport

GeneID:3191101	FTT_1696	groEL	molecular chaperone GroEL. Promotes refolding of misfolded polypeptides especially under stressful conditions	-2.1948149	5.28E-09	Stress response
GeneID:3191298	FTT_1699	pdpA2	hypothetical protein (TSSS)	-2.5056395	7.14E-10	Virulence - FPI
GeneID:3192364	FTT_1701	hypothetical protein		-2.009657	5.02E-05	Hypothetical and unknown function
GeneID:3192355	FTT_1702	hypothetical protein	Similar to AAP58969.1 (Q7XI38) unknown protein from Francisella novicida	-2.4205987	1.98E-08	Hypothetical and unknown function
GeneID:3192362	FTT_1704	hypothetical protein	Similar to AAP58972.1 (Q7X3I5) unknown protein from Francisella novicida	-2.2177311	3.97E-08	Hypothetical and unknown function
GeneID:3192369	FTT_1705	hypothetical protein	Similar to AAP58973.1 (Q7X3I4) unknown protein from Francisella novicida	-2.1445014	1.08E-07	Hypothetical and unknown function
GeneID:3192554	FTT_1706	hypothetical protein	Similar to AAP58974.1 (Q7X3I3) unknown protein from Francisella novicida	-2.2849797	3.89E-08	Hypothetical and unknown function
GeneID:3192534	FTT_1707	hypothetical protein		-1.5299172	0.000171102	Hypothetical and unknown function
GeneID:3192363	FTT_1711 c	iglD2	intracellular growth locus subunit D	-2.6444571	6.07E-11	Virulence - FPI

GeneID:3191814	FTT_1712 c	iglC2	intracellular growth locus subunit C	-2.6919535	5.27E-11	Virulence - FPI
GeneID:3191488	FTT_1713 c	iglB2	intracellular growth locus subunit B	-3.0012097	1.35E-11	Virulence - FPI
GeneID:3191759	FTT_1714 c	iglA2	intracellular growth locus subunit A	-2.5594086	4.65E-10	Virulence - FPI
GeneID:3191313	FTT_1715 c	pdpD2	hypothetical protein (TSSS)	-2.572393	2.55E-10	Virulence - FPI
GeneID:3191766	FTT_1747		Similar to AAO90156 (Q83DT1) Outer membrane protein OmpH, , from Coxiella burnetii	-2.0211511	2.34E-07	Membrane protein
GeneID:3190727	FTT_1752	ssb	single-strand binding protein	-2.0975774	1.29E-07	DNA replication
GeneID:3190860	FTT_1768 c		chitinase	2.14305612	0.000112552	Carbohydrate metabolism
GeneID:3192509	FTT_1769 c	clpB	ClpB protein	-1.8166672	2.89E-06	Stress response
GeneID:3192304	FTT_1771	hypothetical protein		-2.1159618	1.20E-07	Hypothetical and unknown function
GeneID:3190801	FTT_1778 c	hypothetical protein		-1.7273575	4.94E-06	Hypothetical and unknown function
GeneID:3190882	FTT_1783		major facilitator transporter	1.63362667	0.000123049	Transport
GeneID:3191780	FTT_1791	hypothetical protein		-1.4743976	2.59E-05	Hypothetical and unknown function
GeneID:3191327	FTT_1793 c	pepN	aminopeptidase	-1.7235679	1.52E-05	Amino acid biosynthesis
GeneID:3192536	FTT_1794		heat shock protein	-1.872188	4.21E-07	Stress response

GeneID:3192283	FTT_1798c	hypothetical protein		2.00551032	9.25E-07	Hypothetical and unknown function
GeneID:3190959	FTT_r01		5S ribosomal RNA	2.45227929	1.12E-05	Translation
GeneID:3192225	FTT_t04		tRNA-Gly	-1.8422021	0.000252133	Translation
GeneID:3192285	FTT_t06		tRNA-Trp	-1.7359176	0.000129704	Translation
GeneID:3191326				-2.1332388	1.08E-07	Hypothetical and unknown function
GeneID:3191305				-1.8814698	2.21E-06	Hypothetical and unknown function
GeneID:3191803				-1.5308216	0.000137076	Hypothetical and unknown function
GeneID:3191223				1.47443195	0.000159059	Hypothetical and unknown function
GeneID:3191356				1.85207543	1.80E-05	Hypothetical and unknown function
GeneID:3190990				2.02036656	2.15E-06	Hypothetical and unknown function
GeneID:3191855				2.05360014	2.20E-06	Hypothetical and unknown function
GeneID:3192306				2.2208877	4.20E-06	Hypothetical and unknown function
GeneID:3191492				2.27394393	2.15E-06	Hypothetical and unknown function

TABLE S1 The most significantly differentially expressed genes between 0 and 1 µg/mL serine hydroxamate-treated conditions. Genes are arranged by locus tag.

**Primers for RT-PCR**	**Sequence 5'→3'**
iglCrtpcrF	ACAGGTAACAAGTGGCGAGAC
iglCrtpcrR	CTGCGCAACATACTGGCAAAC
16SrtpcrF	AATTGCTATTGCTGGCAGTGAAC
16SrtpcrR	GAGCAGTTTCTGCTTTAAGTATTC
FTT1334F	TTACCACGATAGGTTTGTCTG
FTT1334R	GTTTGCTGGACTAGCTTAGAC
FTT0613F	TAATATCTCTAACTAGTATTG
FTT0613R	TTGGCAGCCAATTGTAATACG

TABLE S2 Primers used in this study.
